# Supplementary material for: Relationship between popularity and the likely efficacy: an observational study based on a random selection on top-ranked physical activity apps
Source: BMJ Open. 2019 Nov 14;9(11):e027536. doi: 10.1136/bmjopen-2018-027536 (PMC6886953; doi:10.1136/bmjopen-2018-027536)
Supplement: Supplementary data [file bmjopen-2018-027536supp001.pdf]

Supplementary file 1: Definition of PA app features with the frequency of occurrence and the number of features per app (n= 65)

	Features	Definitions/examples
1.	PA EDUCATION IN A VARIETY OF FORMATS	Information, instruction, guidance on PA, e.g., written material, image, audio, video instructions
2.	AUTOMATIC TRACKING	Any data automatically recorded by the app, e.g., step count, duration, PA sessions completed
3.	LOGS	Any data inputted by the user, e.g., calories, weight, gym sessions completed
4.	REPORTS	Any reports based on automatically recorded or user-inputted data, e.g., graphs of the number of steps completed in a week, summaries
5.	GAMIFICATION	Gamification was defined as "the use of game design elements in non-game contexts"*, e.g., provision of, medals, badges, points etc.
6.	NOTIFICATIONS	Messages reminding the user to compete a PA session
7.	IN-APP COMMUNITY	Option to communicate with other users, e.g., forums for sharing PA advice; feed with user reporting on PA sessions completed
8.	SHARING ON SOCIAL MEDIA	Option to post information on completed PA session on social media
9.	INTEGRATION WITH OTHER APPS OR	Option to use or connect with other apps or devices, e.g., Apple Health, music library, sensors such as heart rate monitor
10.	TAILORING of PA	Option to change the default setting of PA sessions, e.g., duration, pace, timing of intervals

*Definition of gamification from: Huotari, K. and Hamari, J. Defining gamification: a service marketing perspective. In *Proceeding of the 16th international academic MindTrek conference;* 2012. ACM. doi: 10.1145/2181037.2181040

**App feature (n,%)**		
PA education	Yes	44 (67.7)
	No	21 (32.3)
Automatic Tracking	Yes	61(93.9)
	No	4(6.2)
Logs	Yes	24(36.9)
	No	41(63.1)
Reports	Yes	45(69.2)
	No	20(30.8)
Gamification	Yes	27(41.5)
	No	38(58.5)
Reminders	Yes	31(47.7)
	No	34(52.3)
In-app community	Yes	21(32.3)
	No	44(67.7)
Sharing on social media	Yes	32(49.2)
	No	33(50.8)
Integration with other apps/devices	Yes	35(53.8)
	No	30(46.2)
Tailoring of PA	Yes	57(87.7)
	No	8(12.3)

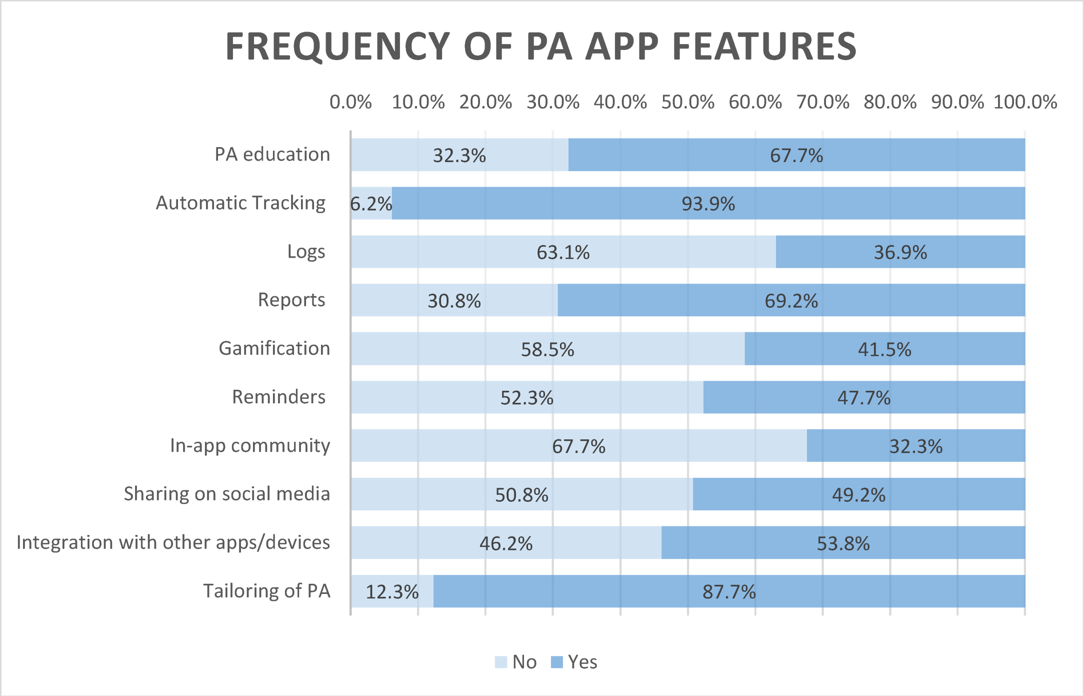

	App name	Number of Features
1.	Fitbit	9
2.	Strava Running and Cycling GPS	8
3.	Pacer – Pedometer plus Weight Loss and BMI Tracker	7
4.	Map My Run – GPS Running & Workout Tracker	9
5.	Adidas train & run	9
6.	Steps Pedometer & Step Counter Activity Tracker	2
7.	7 Minute Workout by Simple Design Ltd	8
8.	Runtastic Running & Fitness	12
9.	Home workout MMA Spartan Free	4
10.	Stepz – Pedometer & Step Counter	9
11.	Interval Timer – Timing for HIIT Training and Workouts	3
12.	Sworkit – Custom Workouts for Exercise & Fitness	7
13.	Fitness & Bodybuilding	5
14.	Daily Workouts FREE	4
15.	30 Day Ab Challenge FREE	6
16.	Runtastic Results: Body Workout Fitness Trainer	9
17.	C25K® - 5K Running Trainer	8
18.	Health Mate – Steps tracker & Life coach	7
19.	One You Couch to 5K	6
20.	Running, Walking and Biking with Endomondo	8
21.	Map My Ride – GPS Cycling & Route Tracker	9
22.	Interval Timer	1
23.	5K Run - Couch to 5K	5
24.	7 Minutes Workout – Women Fitness Exercise Trainer	6
25.	Seconds – Interval Timer	4
26.	Running Distance Tracker +	4
27.	Freeletics Bodyweight – Workout	8
28.	Couch to 10K Running Trainer	8

29.	FitNotes - Gym Workout Log	4
30.	Belly Fat Exercises	1
31.	Belly Fat Workout FREE – 10 Minute Ab Exercises	3
32.	Movesum – Step counter by Lifesum	3
33.	7 Minute Workout Challenge by Fitness Guide Inc	8
34.	Adrian James 6 Pack Abs Workout	6
35.	Full Fitness : Exercise Workout Trainer	5
36.	Runtastic PRO Running and Workout Tracker	10
37.	Couch to 5K® - Running App and Training Coach	9
38.	Adrian James High Intensity Interval Training	6
39.	Running for Weight Loss PRO	9
40.	Fitness Trainer FULL version	4
41.	Instant Fitness : 600+ exercises, 100+ workouts	5
42.	Push ups 0 to 100: push ups challenge trainer pro	7
43.	Couch to 5K Runner, 0 to 5K run training	7
44.	Footsteps – Pedometer	8
45.	iMuscle 2	6
46.	10K Running Trainer Pro	8
47.	Police Fitness – Bleep Test	2
48.	Marathon Trainer - 26.2 42K	8
49.	MapMyFitness+ Workout Trainer	7
50.	5K to 10K	7
51.	7 Minute Workout Pro	7
52.	Chloe Madeley Weights 4 Women	1
53.	10K Pacer: Run pace training. Run faster	8
54.	Starting Strength Official	5
55.	Thor Fitness: 60 Day Bodyweight Workout Routine	3
56.	Half Marathon Trainer 13.1 21K	8
57.	PDC Pole Dance Syllabus	1
58.	MMA Spartan Workouts Pro	5
59.	Get Running (Coach to 5K)	5
60.	WalkJogRun GPS Running Routes	9
61.	Runtastic Road Bike PRO	8
62.	Runtastic Mountain Bike PRO GPS Biking Computer, Trail and Route Tracker	11
63.	Chloe Madeley 15 minute fat loss workout	2
64.	CARROT Fit – 7 Minute Workout, Step Counter Weight Tracker	11
