# Supplementary material for: Relationship between popularity and the likely efficacy: an observational study based on a random selection on top-ranked physical activity apps
Source: BMJ Open. 2019 Nov 14;9(11):e027536. doi: 10.1136/bmjopen-2018-027536 (PMC6886953; doi:10.1136/bmjopen-2018-027536)
Supplement: Supplementary data [file bmjopen-2018-027536supp002.pdf]

Supplementary file 2: Sensitivity analysis including only those BCTs for which there was high level of confidence in the presence of the BCT (++)

**Variable**	**App store**	**OR (CI)**	**App store**	**OR (CI)**
Group 1: Goals and planning	iTunes	1.16 (0.87-1.55)	Google Play	1.11 (0.83-1.49)
1.1. Goal setting (behavior)	iTunes	0.91 (0.47-1.76)	Google Play	1.65 (0.85-3.20)
1.3. Goal setting (outcome)	iTunes	1.18 (0.64-2.18)	Google Play	0.81 (0.44-1.49)
1.4. Action planning	iTunes	1.07 (0.63-1.84)	Google Play	1.42 (0.84-2.40)
Group 2: Feedback and monitoring	iTunes	1.62 (1.22-2.13)*	Google Play	1.25 (0.95-1.64)
2.2. Feedback on behaviour	iTunes	2.07 (1.02-4.22)*	Google Play	1.76 (0.84-3.68)
2.3. Self-monitoring of behaviour	iTunes	1.57 (0.95-2.61)	Google Play	2.05 (1.23-3.39)*
2.4. Self-monitoring of outcome(s) of behaviour	iTunes	1.61 (0.93-2.77)	Google Play	0.98 (0.57-1.69)

Feedback on behavior crosses into significance for iTunes. Otherwise, the results are very consistent with the original findings.
