# Supplementary material for: Relationship between popularity and the likely efficacy: an observational study based on a random selection on top-ranked physical activity apps
Source: BMJ Open. 2019 Nov 14;9(11):e027536. doi: 10.1136/bmjopen-2018-027536 (PMC6886953; doi:10.1136/bmjopen-2018-027536)
Supplement: Author's manuscript [file bmjopen-2018-027536.draft_revisions.pdf]

BMJ Open is committed to open peer review. As part of this commitment we make the peer review history of every article we publish publicly available.

When an article is published we post the peer reviewers' comments and the authors' responses online. We also post the versions of the paper that were used during peer review. These are the versions that the peer review comments apply to.

The versions of the paper that follow are the versions that were submitted during the peer review process. They are not the versions of record or the final published versions. They should not be cited or distributed as the published version of this manuscript.

BMJ Open is an open access journal and the full, final, typeset and author-corrected version of record of the manuscript is available on our site with no access controls, subscription charges or pay-per-view fees (http://bmjopen.bmj.com).

If you have any questions on BMJ Open's open peer review process please email info.bmjopen@bmj.com

BMJ Open

Relationship between popularity and the likely efficacy of publicly available physical activity apps

Journal:	*BMJ Open*
Manuscript ID	bmjopen-2018-027536
Article Type:	Research
Date Submitted by the Author:	31-Oct-2018
Complete List of Authors:	Bondaronek, Paulina; University College London, eHealth Unit, Research Department of Primary Care and Population Health<br>Slee, April; University College London, Research Department of Primary Care and Population Health<br>Hamilton, Fiona; University College London, eHealth Unit, Research Department of Primary Care and Population Health<br>Murray, Elizabeth; University College London, Primary Care and Population Health
Keywords:	mHealth, PUBLIC HEALTH, Physical Activity, Behaviour Change, Intervention

SCHOLARONE™
Manuscripts

Relationship between popularity and the likely efficacy of publicly available physical activity apps

Bondaronek Paulina [1], Slee April [2], Hamilton Fiona [1], Murray Elizabeth [1]

1 - eHealth Unit, Research Department of Primary Care and Population Health, University College London
2 - Research Department of Primary Care and Population Health, University College London

Corresponding author: Paulina Bondaronek, eHealth Unit, Department of Primary Care & Population Health, University College London, Upper Third Floor, UCL Medical School (Royal Free Campus) Rowland Hill Street, London NW3 2PF; email: p.bondaronek@ucl.ac.uk; phone: 07742966769

Word count: 4098

For peer review only

ABSTRACT

Objectives: To explore the relationship between popularity of mobile application (apps) for physical activity (PA) and their likely efficacy. The primary objective was to assess the association between app popularity (indicated by user ratings) and likely efficacy (indicated by the number of Behaviour Change Techniques, BCTs, present). The secondary objective was to assess the relationship between user ratings and those BCTs that have been shown to be effective in increasing PA.

Methods and analysis: 400 top-ranked free and paid apps from iTunes and Google Play (GP) stores were screened, and were included if (i) the primary behaviour targeted was PA; (ii) they had stand-alone functionality; and (iii) they targeted adults. The outcome variable of user rating was dichotomised into high (4,5 stars) or low (1,2,3 stars) ratings. Logistic regression models were performed to determine the association between the presence of BCTs and user ratings.

Results: Of 400 apps, 156 were eligible and 65 apps were randomly selected, downloaded and assessed by two reviewers. There was no relationship overall between star ratings and the number of BCTs present, nor between star ratings and the presence of BCTs known to be effective in increasing PA. App store was strongly associated with star ratings, with iTunes users less likely to give 4 or 5 stars compared to GP users (Odds ratio, OR: 0.74 95% CI: 0.73-0.76, $p < 0.001$).

Conclusions: This study suggests that popularity does not necessarily imply the likelihood of effectiveness. Hence, public health impact is unlikely to be achieved by allowing market forces to “prescribe” what is used by the public.

For peer review only

Strengths and limitations of this study

- First study to assess the relationship between popularity of physical activity apps and their likely efficacy in two major app stores
- A systematic approach to sample identification and assessment including a standardised assessment tool used to characterise behaviour change interventions
- Sample identified and assessed by two independent reviewers
- It is unknown what variables comprise the ranking algorithm from which the sample was derived
- It is possible that user ratings, as expressed by the stars assigned to the apps, can relate to different aspects of the app content and functionalities

Keywords: mHealth, PUBLIC HEALTH, Physical Activity, Behaviour Change, Intervention

For peer review only

For peer review only - http://bmjopen.bmj.com/site/about/guidelines.xhtml

INTRODUCTION

The accessibility, convenience, and wide reach of apps create new avenues for health behaviour change on a large scale. Out of the total 325,000 health apps available on the market in 2017 [1] the largest app groups were fitness apps. Yet, 28.7% of adults in England are inactive [2] and physical inactivity increases the risk of up to 20 long-term health conditions [3]. The rapidly increased market supply of the apps reflects public demand for the new means of engaging in health behaviours and 34% percent of mobile phone owners have at least one health-related app downloaded on their mobile phone [4].

Research assessing the relationship between the popularity of apps and their quality has found mixed results. Azar, et al. [5] found that apps for weight management that were of higher quality, defined as inclusion of the constructs from four behaviour change theories, were not among the highest ranked apps the app stores. Similarly, apps that had higher download rates or higher ranking were associated with less adherence to guidelines in smoking cessation apps [6]. On the other hand, Pereira-Azevedo, et al. [7] reviewed 129 urology apps in GP app store and found that higher download rates were associated with expert involvement in the development of the apps. These studies exploring the relationship between popularity and quality in apps need to be interpreted with caution. They targeted different health conditions and behaviours, and used different definitions of quality, such as consistency with behaviour change theory, expert involvement, and adherence to guidelines. None of these studies used behaviour change theory to systematically assess the content of the apps in terms of their likely efficacy and how it relates to app popularity.

The Behaviour Change Technique (BCT) Taxonomy [8] is a systematic method used to specify the content of behaviour change interventions [9]. The Taxonomy has also been used to quantify the inclusion of behaviour change theory in interventions, including apps [10-12]. For example, Bardus, et al. [10] extracted BCTs from 23 apps aimed at weight management. They used the Mobile App Rating Scale (MARS) to assess the quality of apps [13] and showed a positive association between the number of BCTs and the MARS subsets: Engagement, Functionality, and Aesthetics, as well as the overall quality of app score. This may suggest that the inclusion of BCTs may be related to the quality of the apps, as assessed by MARS.

However, the same authors found no association between user ratings and either BCTs or the quality indicators on the MARS scale suggesting that apps that were highly rated were not of high quality. Similarly, Crane, et al. [11] showed that only 1 BCT, "prompt review of goals", was associated with user ratings in alcohol reduction apps. The authors concluded that there was little association between the mention of theory, BCTs inclusion and popularity of the alcohol reduction apps. These findings indicate that further work is needed to explore whether popular apps are those that are of high quality and are likely to be effective. This has relevance for public health policy as the combination of popularity with likely efficacy would suggest that apps have a potential role in promoting public health, whereas a disconnect between popularity and likelihood of efficacy would raise questions about leaving the market to guide user choices in app downloads.

Systematic reviews and meta-analyses have shown that self-regulation techniques, such as goal setting, monitoring, and feedback are effective in increasing PA [14-17] behaviour and they may have cumulative effects. For example, Michie, et al. [15] showed that self-monitoring with other self-regulatory BCTs were more effective in increasing PA using one of those BCTs in isolation. Self-regulation has been acknowledged as important constructs in behaviour change theories, e.g. in Control Theory [18] and Self-Regulation Theory [19]. Hence, the presence of these BCTs can be used as an indicator for quality of those apps and a proxy measure of their likely efficacy.

Aims and objectives

The aim of this study was to explore the relationship between the user ratings as a marker of popularity of apps and the inclusion of BCTs as a marker of likely efficacy in publicly available PA apps. Specifically, the primary objective was

For peer review only - http://bmjopen.bmj.com/site/about/guidelines.xhtml

to assess the association between user ratings and the number of BCTs included in the sample of popular PA apps. The secondary objective assessed the inclusion of BCTs shown to be effective in increasing PA behaviour, in particular BCTs related to self-regulation of behaviour.

METHODS

Design

The study used a random sample of popular apps to determine the association between user ratings and the presence of BCTs.

Data sources and collection

The sample included popular PA apps identified from 400 top-ranked free and paid apps from the Health and Fitness category available in the UK version of iTunes and GP app stores on 17/10/16. Apps were included if (i) the primary behaviour targeted was PA; (ii) they had stand-alone functionality; and (iii) they targeted adults. Two reviewers (PB, GA) reviewed each app, assessed whether specific BCTs were present, and extracted relevant data. The detailed process of app identification and sample description is provided in Bondaronek, et al. [20].

Data extraction

Descriptive data included the cost and size of the app; the number and distribution of star ratings; and the presence of BCTs.

BCTs extraction as a measure of likely efficacy

The presence or absence of 93 BCTs in the BCT Taxonomy v1 as described by Michie, et al. [8] were classified using 3 categories: absent, appears to be present but evidence is not clear (+), present beyond doubt and evidence clear (++). The presence of self-regulation BCTs (associated with PA intervention effectiveness) was classified using the BCT Taxonomy Grouping 1: Goal and planning, and Grouping 2: Feedback and monitoring.

User ratings

The average star rating was calculated by summing the number of stars awarded across all users and dividing them by the number of users that submitted the rating. When an app appeared in both stores, a weighted average of the star ratings for each store was calculated based on the relative proportion of the ratings in each store. This algorithm is equivalent to summing the number of stars awarded by all users in both stores and then dividing them by the total number of reviews across both stores. This calculation weights users equally regardless of the platform used to access the app.

Variables determined a priori potentially to affect the relationship between BCTs and higher or lower ratings were app store (iTunes or GP), number of features, whether the app was free or required payment, size (in megabytes, MB) and usability.

Number of features

Health apps use technology-enhanced features to deliver BCTs in order to influence behaviour. The apps were categorised according to the features offered by the app, e.g., PA tracking, reminders, app community, data visualisation, etc.). To the authors' best knowledge, at the time of writing, there was no standardised list of features that are commonly used in PA apps. The first ten apps extracted were used to compile a list of PA app features. The list was continuously updated throughout the app extraction process in order to accommodate for new features that were found in the PA app sample.

For peer review only - http://bmjopen.bmj.com/site/about/guidelines.xhtml

Usability

Usability was assessed using the System Usability Scale, SUS, [21] which consists of 10 items ranked on a 5-point Likert scale, from “strongly disagree” to “strongly agree”. To make the wording of the scale more applicable to the study, two changes were made: 1) the wording of the 8th statement was changed from "cumbersome" to "awkward" as recommended [22-24]; 2) the word "system" was replaced by "app”. SUS yields scores from 0-100 where higher scores indicate greater usability. The interpretation of the SUS score used the thresholds proposed and validated by Bangor, et al. [22] with 72.5 described as good usability.

Data analysis

Agreement statistics including the prevalence and bias adjusted kappa statistic, PABAK [25] and unadjusted kappa [26] were calculated. Disagreements were then resolved by discussions among the two reviewers and consultation with other authors if unresolved.

The number of BCTs in the apps was summarized using the mean, standard deviation, median, 25th and 75th percentiles, and the maximum and minimum. Similar statistics were used to summarize user ratings, cost, size, and SUS score. The summary descriptive tables were presented for free and paid apps separately and in total as app stores have separate rankings based on the cost.

The pre-specified primary analysis was based on a linear regression of the number of BCTs on star average as this continuous outcome required the smallest sample of apps for adequate statistical power. Logistic regression to determine the relationship between the number of BCTs and the odds of a 4 or 5 star rating (versus a 1, 2 or 3 star rating) was the pre-specified secondary analysis.

Sample size calculations

A pilot study based on 10 apps suggested that mean star ratings were normally distributed. Hence, a sample size calculation was undertaken using mean ratings as a continuous measure in a linear model. Based on this framework, a sample size of 51 apps would provide 90% power to detect a change of 0.11 for each additional BCT at 5% significance level (type I error rate). A sample size of 65 apps was selected to account for any randomly selected apps that might not fulfil the inclusion criteria once downloaded. However, the pilot sample was not sufficiently large enough to show the skew in star average for the complete sample, which would preclude the use of linear models. The difference in star rating by store was also unknown at the time of study planning, and hence the app store interactions were a post-hoc addition to this plan. A retrospective power analysis was conducted. Based on simulations, this study had 97% power to detect an OR >= 1.2 with 64 apps. Since we had high power and did not see a significant result, this suggests that the true increase in the chance of a star rating of 4 or 5 with each additional BCT has an OR of < 1.2.

As indicated in the analysis plan, star ratings of 4 and 5 were classified as high ratings, while ratings of 1, 2 and 3 were classified as low ratings. Modelling proceeded in a series of steps. First, we tested a small set of variables identified in the analysis plan for the relationship to star rating (cost, usability, number of features, size) by including each of these as fixed covariates in univariate logistic regression models with the outcome of high versus low ratings. Any variables significant at $p \leq 0.1$ were included in models examining BCTs. App store and the app store-by-BCT interaction terms were included in each model to reflect that the clientele for iTunes and GP may be different, and that these differences may impact the relationship between BCTs and star ratings. Weighted logistic regression (by the number of responses for each app) was used with a random intercept term for app, reflecting that the apps selected for analysis were a random sample of available fitness apps, and to control for correlation when the same app was reviewed in both stores. Analyses were performed using SAS version 9.3 and R version 3.3.3.

Sensitivity analysis

Sensitivity analyses included a revision of the number of BCTs including only those that were present beyond any doubt (++) [27], and dichotomising user ratings into 5 stars versus < 5 stars.

For peer review only - http://bmjopen.bmj.com/site/about/guidelines.xhtml

Patient and Public Involvement

This research was conducted without patient involvement.

RESULTS

Sample

Out of 400 apps, 244 apps were excluded (see Figure 1). Of the remaining 156 PA apps, 31 were duplicates in that the same app appeared in both iTunes and GP. Thus, 125 unique apps were eligible for random selection; 65 unique apps were randomly selected for analysis including 32 free apps and 33 paid apps. One app, Yoga Break, was excluded from analyses of user reviews as the data on user ratings was not available due to the small number of ratings, resulting in a final sample of 64 PA apps. These apps collectively received more than 2.8 million user ratings.

Figure 1: Flow chart of the apps included in the analysis

<Figure 1 separate file>

There was substantial agreement between the reviewers in the assessment of BCTs (PABAK =0.94, 95% CI 0.93 to 0.95, Kappa =0.78, 95% CI 0.75 to 0.81).

Characteristics of the apps are displayed in Table 1. The number of BCTs and features were approximately normally distributed, with the mean number of BCTs being 7.1 (SD= 3.0) and the mean number of features being 6.3 (SD=2.7). Star ratings, size, SUS score and the cost variables were skewed. The median star rating was 4.5 (IQR 1.9 – 4.9) and the median SUS score was 86.3 (IQR 75.00 – 91.88). Among paid apps, the median cost was £2.40 (IQR 1.78 – 2.99).

Table 1: Characteristics of PA apps included in the sample

		**Free (N=32)**	**Paid (N=32)**	**Total (N=64)**
Number of BCTs	Mean ± SD	6.56 ± 2.99	7.56 ± 2.87	7.06 ± 2.95
	Median	7.00	8.00	8.00
	25, 75%tile	5.00, 8.00	6.00, 10.00	5.00, 9.00
	Min, Max	1.0, 12.0	1.0, 13.0	1.0, 13.0
Star average*	Mean ± SD	4.26 ± 0.65	4.35 ± 0.45	4.30 ± 0.56
	Median	4.50	4.49	4.50
	25, 75%tile	4.30, 4.60	4.29, 4.60	4.30, 4.60
	Min, Max	1.9, 4.7	3.0, 4.9	1.9, 4.9
Number of Features	Mean ± SD	6.13 ± 2.73	6.44 ± 2.66	6.28 ± 2.68
	Median	6.50	7.00	7.00
	25, 75%tile	4.00, 8.00	5.00, 8.00	4.00, 8.00
	Min, Max	1.0, 12.0	1.0, 11.0	1.0, 12.0

For peer review only - http://bmjopen.bmj.com/site/about/guidelines.xhtml

		Free (N=32)	Paid (N=32)	Total (N=64)
Size (MB)*	Mean ± SD	45.53 ± 40.85	61.51 ± 47.22	53.52 ± 44.53
	Median	32.45	53.97	39.64
	25, 75%tile	16.82, 62.27	28.92, 85.02	25.52, 79.50
	Min, Max	1.9, 163	1.0, 242	1.0, 242
Usability (SUS)	Mean ± SD	81.25 ± 12.64	85.23 ± 12.02	83.24 ± 12.40
	Median	85.00	87.50	86.25
	25, 75%tile	71.88, 91.25	78.75, 93.75	75.00, 91.88
	Min, Max	52.5, 100	57.5, 100	52.5, 100
Cost (GBP) *	Mean ± SD		3.00 ± 2.12	
	Median	N/A	2.35	N/A
	25, 75%tile		1.78, 2.99	
	Min, Max		0.8, 9.0	

*weighted average of iTunes and Google Play; GBP: British pounds, N/A, not applicable

User ratings

In total, 2,819,469 user ratings of the 64 apps were used in the analysis. Among these, 88.5% were 4 or 5 star reviews and 11.5% were 1, 2 or 3-star reviews. Among the covariates considered for model inclusion, good usability increased the chance of a 4 or 5 star rating compared to poor usability (OR: 1.66, 95% CI 0.96 to 2.89, p=0.071). This covariate was included in all subsequent models. App store was strongly associated with star ratings with iTunes users less likely to give 4 or 5 stars compared to GP users (OR: 0.74 95% CI: 0.73-0.76, p< 0.001). Further investigation of the difference between the stores and user ratings showed that users in GP were 37% more likely to award 4 or 5 compared to those in iTunes (95%CI 35% to 41%, p < 0.001). A similar pattern can be seen comparing the weighted average within-app star rating across stores. There were 17 apps that were sold in both stores (Figure 2). Analysis was conducted to assess whether the proportion with the high star ratings in GP exceeds what would have been found by chance. If there were no relationship between store and star rating, we would expect the star rating in iTunes to exceed the star rating in GP for about 50% of apps (coin flip). Mean star rating in GP exceeded the rating in iTunes in 13/17 apps (76.5%, 95% CI: 64.2-86.2, p<0.0001). Since the analysis was significant, we can rule out that the difference between the user ratings in the stores was due to chance. Cost, size and number of features were not related to star ratings.

App store was strongly associated with star ratings with iTunes users less likely to give 4 or 5 stars compared to GP users (OR: 0.74 95% CI: 0.73-0.76, p< 0.001). Further investigation of the difference between the stores and user ratings showed that users in GP were 37% more likely to award 4 or 5 compared to those in iTunes (95%CI 35% to 41%, p < 0.001). A similar pattern can be seen comparing the weighted average within-app star rating across stores. There were 17 apps that were sold in both stores (Figure 2). Analysis was conducted to assess whether the proportion with the high star ratings in GP exceeds what would have been found by chance. If there were no relationship between store and star rating, we would expect the star rating in iTunes to exceed the star rating in GP for about 50% of apps (coin flip). Mean star rating in GP exceeded the rating in iTunes in 13/17 apps (76.5%, 95% CI: 64.2-86.2, p<0.0001).

For peer review only - http://bmjopen.bmj.com/site/about/guidelines.xhtml

The significance of this p-value implies that the chance that a higher score in GP is the same as the chance of a high score in iTunes.

Primary analysis

The primary analysis of the association between user ratings and the overall number of BCTs included in each app found no relationship between number of BCTs and star rating. Subgroup analysis showed that in iTunes only, a higher star rating was associated with the total number of BCTs. (OR: 0.05, 95%CI: 0.97-1.14, p = 0.236).

Figure 2: The comparison of star average between the same apps that existed in both stores (n=17)

<Figure 2 separate file>

Based on the model containing the store interaction (Figure 3), in iTunes there was an association with each additional BCT corresponding to 15% increase in the chance of a higher star rating (OR: 1.15, 95% CI: 1.06, 1.25), there was no association between the number of BCTs and GP. Usability was not significant in the multivariate model (OR 1.57, 95% CI: 1.91, 2.71)

Figure 3: ORs for the associations between the number of BCTs and a 4- or 5- star rating

<Figure 3 separate file>

Secondary objective

The secondary analysis examined the associations between a 4- or 5- star user rating and the inclusion of BCTs that have been shown to be effective in increasing PA behaviour.

The frequency of the BCTs associated with efficacy for increasing PA behaviour is presented in Figure 4.
The BCT grouping 1 (Goal and planning) was represented in 84.4% of evaluated apps. The most common of these was goal setting for behaviours (84.4%), followed by action planning (35.9%) and goal setting for outcomes (18.8%). The BCT grouping 2 (Feedback and monitoring) was included in 92.2% of evaluated apps. Feedback on behaviour was included in nearly all apps (90.6%), while self-monitoring of behaviour and of outcomes were incorporated in about a third of apps.

Figure 4: Frequency of individual BCTs within the two Groupings of self-regulatory BCTs

<Figure 4 separate file>

The number of Grouping 1 BCTs had no impact on star rating in either store (Figure 5). Among iTunes users, each additional BCT from Grouping 2 increased the chance of a high star rating by 63% (OR: 1.63 95% CI: 1.23-2.16*).* There was no association between the number of BCTs and star rating among GP users. Self-monitoring of behaviour was associated with higher ratings in GP (OR: 2.05, 95% CI: 1.23-3.39)

Figure 5: ORs for the associations between BCTs that have been shown to be effective and a 4- or 5- star rating
<Figure 5 separate file>

For peer review only - http://bmjopen.bmj.com/site/about/guidelines.xhtml

For peer review only

Sensitivity analysis

The sensitivity analyses including only those BCTs that were classified as "present beyond all reasonable doubt" [27] showed that for iTunes, the BCT 2.2 Feedback on behaviour crossed into significance. Otherwise, the results are consistent with the original findings. The results of the sensitivity analysis are available in the Supplementary file 1.

DISCUSSION

This study assessed the relationship between the popularity of publicly available PA apps (assessed through user ratings) and their likely efficacy (assessed through the inclusion of BCTs). Overall for both app stores, there was no association between popularity and likely efficacy as indicated by the overall presence of BCTs and the BCTs known to be effective in increasing PA. However, users in each app stores differed and there was an association between the number of BCTs and high user rating in iTunes but not in GP.

This study supports the findings of previous research in apps targeting weight management [5 10], smoking cessation [6] and alcohol reduction [11] which showed that apps that were highly rated, highly ranked or highly downloaded were not necessarily of high quality. The finding of an association between user rankings and overall number of BCTs in one store (iTunes) but not the other (Google Play) is new. There are various possible explanations for this finding, including demographic differences in the population of users of each store [28 29]; differences in the review and approval processes between the two stores [30], or the play of chance. Future studies should explore this observation further, and determine whether it holds for health apps targeting different behaviours.

Strengths

The main strength of the study includes the systematic assessment of the app content conducted by two reviewers. The sample was identified from the most popular publicly available apps from two major app distribution platforms. The use of BCT taxonomy provides a standardised assessment tool, which has been utilised in other studies assessing the content of apps [12 31-33]. Second, Guzman [34] argued that the star rating represents an average score for the whole app that combines both positive and negative evaluations aggregated across users. The study, however, used rater-level data which included individual ratings from 2.8 million users. These large numbers mitigate the problem posed by averaging the star rating across users.

Limitations

This study has some important limitations. First, the main limitation of the study relates to the variables used in this study. It is possible that user ratings, as expressed by the stars assigned to the apps, can relate to different aspects of the app functioning and content. There is evidence suggesting that app reviews tend to occur near a new release which can suggest that the ratings may include comments on the specific updates of the software [35]. In addition, the possibility that user ratings were influenced by fake reviews cannot be excluded [36 37]. However, the user rating was considered the most appropriate measure to explore since it represents a user-led feedback that reflects user experience. Similarly, the choice of the BCTs as an approximation for likely efficacy was selected because studies assessing the efficacy of the apps on the market are scarce [38-40]. Second, the ranking algorithms from which the sample was derived is unknown. Hence, this lack of transparency prevented evaluation of how the calculation of rank might have influenced the app selection. However, apps appear in rank order by default in app stores hence the rank affects what users are seeing. As this study was assessing the most popular apps, the choice of highly ranked apps was considered the most appropriate for the context of this research. Third, GP market tend to have more ratings than iTunes because the process of app review is more complex in the latter [41]. This was addressed in the study by using the weighted averages of the ratings across the stores and by controlling for store in the regression models. While there is a difference in the app review process in both app stores, feedback from both stores should be recognised as

For peer review only - http://bmjopen.bmj.com/site/about/guidelines.xhtml

valid and important. Last, failure to detect the skew in the original primary outcome used to power the study is a limitation, however, a retrospective power analysis showed we had high power to detect an OR of 1.2 and did not find significant result, hence the true OR is likely to be < 1.2.

Implications

This study showed no evidence of association between popularity and likely efficacy. The findings suggest that, at present, allowing the commercial market to determine which PA apps are downloaded is unlikely to be an effective method of public health promotion in terms of increasing overall levels of PA.

Further research is needed to understand which BCTs, and in which combination, are most effective in increasing PA when delivered via an app; how these BCTs can best be delivered; and how to combine features which promote efficacy with those that promote popularity. Last, the differences in iTunes and GP users is an unexpected finding, and not one that the study set out to identify, (i.e. not an a priori hypothesis). Future researchers should be aware of the potential differences between iTunes and GP users and ensure research is carried out on both platforms.

The findings have some implications for public health policy. Apps aimed to increase PA represent the largest category in the two major app stores [42] which illustrates public demand for engaging in PA. The lack of quality in those apps provides a missed opportunity to increase health at the population level. Initiatives to identify and promote high quality apps are in development, e.g., the NHS Digital Tools Library.

Conclusion

To date, this is the first study to assess the association between popularity (measured using user ratings of the apps) and likely efficacy (measured using the inclusion of the BCTs) of publicly available highly-ranked PA apps available in the major app stores. No relationship was found between popularity and likely efficacy suggesting that popularity does not assure high quality, and what is liked may not be what is likely to be effective. However, PA apps in this study were highly rated, highly-ranked apps from the major app stores, hence highly visible for the potential user. Hence, promotion of public health is unlikely to be achieved by allowing market forces to determine which PA apps are used. More studies are needed to assess the effectiveness of apps with users in a real-world setting to investigate the app components that are both, effective and valued by the users.

Supplementary file

Sensitivity analysis including BCTs with only high level of confidence in the presence in the apps (++).

Competing Interests

None declared.

Data Sharing Statement

All data used in the study is available upon request.

Contributorship statement

PB designed the study, collected the data, analysed the data and wrote the manuscript. AS analysed the data and co-wrote the manuscript. EM and FH provided advice on study design, data analysis and interpretation, and contributed to the critical appraisal of the manuscript.

Acknowledgements

We wish to acknowledge Ghadah Alkhaldi (Community Health Sciences Department, College of Applied Medical Sciences, King Saud University, Riyadh, Saudi Arabia) for her help with BCT extraction.

For peer review only - http://bmjopen.bmj.com/site/about/guidelines.xhtml

Funding

PB is a PhD student funded by the Medical Research Council.

References

1. research2guidance. mHealth App Economics: Current Statu and Future Trends in Mobile Health: research2guidance; 2017 [Available from: https://www.uzelf.org/wp-content/uploads/2017/12/R2G-mHealth-Developer-Economics-2017-Status-And-Trends.pdf accessed 20/09/18 2018.
2. Public Health England. Public Health Outcomes Framework: Physical Activity 2016 [Available from: http://www.phoutcomes.info/search/physical%20activity#pat/6/ati/102/par/E12000004.
3. UKActive. Turning the tide of inactivity. *London: UK Active Retrieved* 2014;1(9):15.
4. Terry K. Number of health apps soars, but use does not always follow 2015 [Available from: http://www.medscape.com/viewarticle/851226.
5. Azar KM, Lesser LI, Laing BY, et al. Mobile applications for weight management: theory-based content analysis. *Am J Prev Med* 2013;45(5):583-9. doi: 10.1016/j.amepre.2013.07.005
6. Abroms LC, Padmanabhan N, Thaweethai L, et al. iPhone apps for smoking cessation: a content analysis. *American journal of preventive medicine* 2011;40(3):279-85.
7. Pereira-Azevedo N, Osório L, Cavadas V, et al. Expert involvement predicts mHealth app downloads: multivariate regression analysis of urology apps. *JMIR mHealth and uHealth* 2016;4(3) doi: 10.2196/mhealth.5738
8. Michie S, Richardson M, Johnston M, et al. The behavior change technique taxonomy (v1) of 93 hierarchically clustered techniques: building an international consensus for the reporting of behavior change interventions. *Annals of behavioral medicine* 2013;46(1):81-95. doi: 10.1007/s12160-013-9486-6
9. Michie S, Atkins L, West R. The behaviour change wheel. *A guide to designing interventions 1st ed Great Britain: Silverback Publishing* 2014
10. Bardus M, van Beurden SB, Smith JR, et al. A review and content analysis of engagement, functionality, aesthetics, information quality, and change techniques in the most popular commercial apps for weight management. *Int J Behav Nutr Phys Act* 2016;13:35. doi: 10.1186/s12966-016-0359-9
11. Crane D, Garnett C, Brown J, et al. Behavior change techniques in popular alcohol reduction apps: content analysis. *J Med Internet Res* 2015;17(5):e118. doi: 10.2196/jmir.4060
12. Middelweerd A, Mollee JS, van der Wal CN, et al. Apps to promote physical activity among adults: a review and content analysis. *Int J Behav Nutr Phys Act* 2014;11(1):97.
13. Stoyanov SR, Hides L, Kavanagh DJ, et al. Mobile app rating scale: a new tool for assessing the quality of health mobile apps. *JMIR Mhealth Uhealth* 2015;3(1):e27. doi: 10.2196/mhealth.3422
14. Greaves CJ, Sheppard KE, Abraham C, et al. Systematic review of reviews of intervention components associated with increased effectiveness in dietary and physical activity interventions. *BMC public health* 2011;11(1):119.
15. Michie S, Abraham C, Whittington C, et al. Effective techniques in healthy eating and physical activity interventions: a meta-regression. *Health Psychology* 2009;28(6):690. doi: 10.1037/a0016136
16. Conn VS, Hafdahl AR, Mehr DR. Interventions to increase physical activity among healthy adults: meta-analysis of outcomes. *American journal of public health* 2011;101(4):751-58.
17. Pearson ES. Goal setting as a health behavior change strategy in overweight and obese adults: a systematic literature review examining intervention components. *Patient education and counseling* 2012;87(1):32-42. doi: 10.1016/j.pec.2011.07.018
18. Carver CS, Scheier MF. Control theory: A useful conceptual framework for personality–social, clinical, and health psychology. *Psychological bulletin* 1982;92(1):111.
19. Kanfer FH. Implications of a self-regulation model of therapy for treatment of addictive behaviors. Treating addictive behaviors: Springer 1986:29-47.
20. The public health potential of the current health apps for increasing physical activity. Proceedings of the 2017 International Conference on Digital Health; 2017. ACM.
21. Brooke J. SUS-A quick and dirty usability scale. *Usability evaluation in industry* 1996;189(194):4-7.
22. Bangor A, Kortum PT, Miller JT. An empirical evaluation of the system usability scale. *Intl Journal of Human–Computer Interaction* 2008;24(6):574-94. doi: 10.1080/10447310802205776

For peer review only - http://bmjopen.bmj.com/site/about/guidelines.xhtml

23. Finstad K. The system usability scale and non-native english speakers. *Journal of usability studies* 2006;1(4):185-88.
24. Lewis JR. Psychometric evaluation of the PSSUQ using data from five years of usability studies. *International Journal of Human-Computer Interaction* 2002;14(3-4):463-88. doi: 10.1080/10447318.2002.9669130
25. Byrt T, Bishop J, Carlin JB. Bias, prevalence and kappa. *Journal of clinical epidemiology* 1993;46(5):423-29. doi: 10.1016/0895-4356(93)90018-V
26. Cohen J. Kappa: Coefficient of concordance. *Educ Psych Measurement* 1960;20(37)
27. Wood CE, Richardson M, Johnston M, et al. Applying the behaviour change technique (BCT) taxonomy v1: a study of coder training. *Translational behavioral medicine* 2014;5(2):134-48.
28. Götz FM, Stieger S, Reips U-D. Users of the main smartphone operating systems (iOS, Android) differ only little in personality. *PloS one* 2017;12(5):e0176921.
29. Monetate. Ecommerce Quarterly 2016: Mobile ate the world and is still hungry 2017 [Available from: http://info.monetate.com/rs/092-TQN-434/images/Monetate-EQ3-2016.pdf?mkt_tok=eyJpIjoiTURFMFptTTNNR1prWWpRMyIsInQiOiJsaDE4bXF1OTA4R0o4T0xjNGZjXC9JRkcxcHM4WkRTZFZkT2tnWWorMGtIc24zSUFsanN2Z2tOVDJHTDZFc3F1eTZnZEZJaWJsQUxzQWRjN3F6ZXVNc2wrcVREMDFJc3RMQU5yc0NGdXU4OG41Zk9Gdk1OaGZRUXNEcisreWFEczcifQ%3D%3D accessed 21/09/18 2018.
30. Why Do Users Install and Delete Apps? A Survey Study. International Conference of Software Business; 2017. Springer.
31. Direito A, Dale LP, Shields E, et al. Do physical activity and dietary smartphone applications incorporate evidence-based behaviour change techniques? *BMC Public Health* 2014;14(1):1.
32. Chen J, Cade JE, Allman-Farinelli M. The Most Popular Smartphone Apps for Weight Loss: A Quality Assessment. *JMIR Mhealth Uhealth* 2015;3(4):e104. doi: 10.2196/mhealth.4334
33. Yang CH, Maher JP, Conroy DE. Implementation of behavior change techniques in mobile applications for physical activity. *Am J Prev Med* 2015;48(4):452-5. doi: 10.1016/j.amepre.2014.10.010
34. How do users like this feature? a fine grained sentiment analysis of app reviews. Requirements Engineering Conference (RE), 2014 IEEE 22nd International; 2014. IEEE.
35. User feedback in the appstore: An empirical study. Requirements Engineering Conference (RE), 2013 21st IEEE International; 2013. IEEE.
36. AppAware: which mobile applications are hot? Proceedings of the 12th international conference on Human computer interaction with mobile devices and services; 2010. ACM.
37. Trusting smartphone apps? To install or not to install, that is the question. Cognitive Methods in Situation Awareness and Decision Support (CogSIMA), 2013 IEEE International Multi-Disciplinary Conference on; 2013. IEEE.
38. Direito A, Carraça E, Rawstorn J, et al. mHealth technologies to influence physical activity and sedentary behaviors: Behavior change techniques, systematic review and meta-analysis of randomized controlled trials. *Annals of behavioral medicine* 2017;51(2):226-39.
39. Buechi R, Faes L, Bachmann LM, et al. Evidence assessing the diagnostic performance of medical smartphone apps: a systematic review and exploratory meta-analysis. *BMJ open* 2017;7(12):e018280.
40. Jake-Schoffman DE, Silfee VJ, Waring ME, et al. Methods for evaluating the content, usability, and efficacy of commercial mobile health apps. *JMIR mHealth and uHealth* 2017;5(12)
41. Xu W, Liu Y. mHealthApps: a repository and database of mobile health apps. *JMIR mHealth and uHealth* 2015;3(1):e28. doi: 10.2196/mhealth.4026
42. research2guidance. mHealth app developer economics. The state of the art of mHealth application publishing: research2guidance 2014 [updated 5 May 2016. Available from: http://research2guidance.com/product/mhealth-app-developer-economics-2014/.

For peer review only - http://bmjopen.bmj.com/site/about/guidelines.xhtml

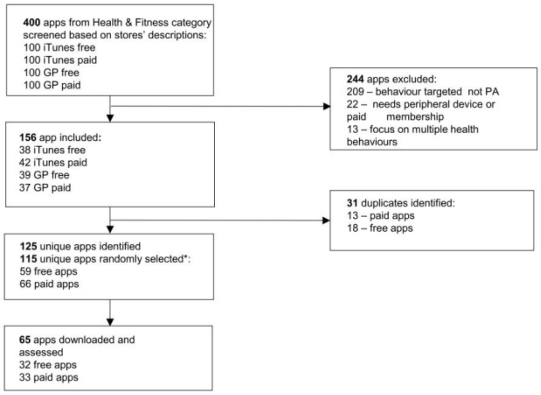

Notes: GP, Google Play; * 10 apps were assessed prior to conduct sample size calculations, hence 115 were randomly selected for the sample

Figure 1: Flow chart of the apps included in the analysis

140x96mm (300 x 300 DPI)

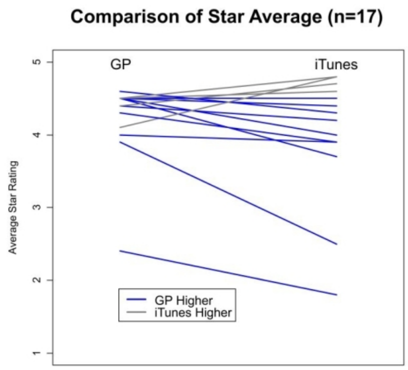

Figure 2: The comparison of star average between the same apps that existed in both stores (n=17)

140x78mm (300 x 300 DPI)

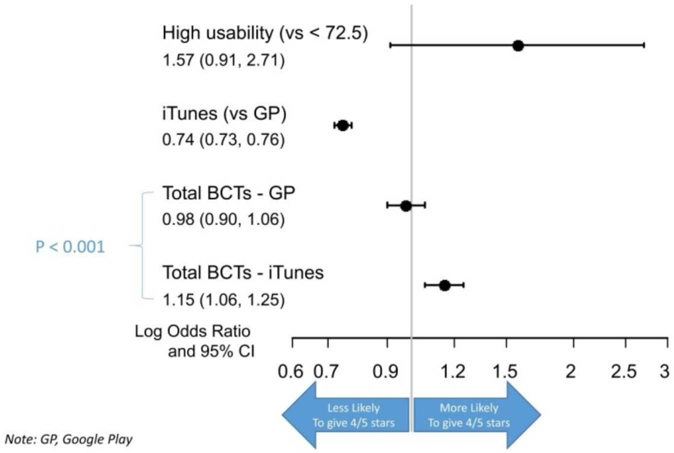

Figure 3: ORs for the associations between the number of BCTs and a 4- or 5- star rating

140x78mm (300 x 300 DPI)

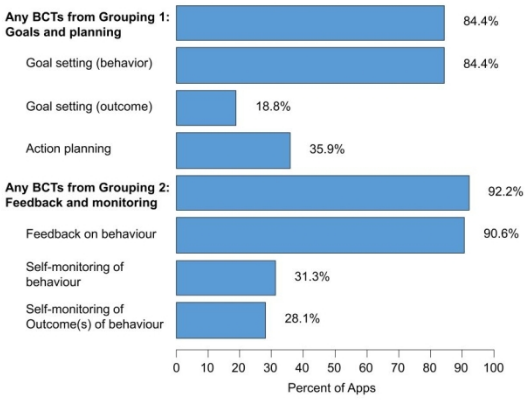

Figure 4: Frequency of individual BCTs within the two Groupings of self-regulatory BCTs

140x78mm (300 x 300 DPI)

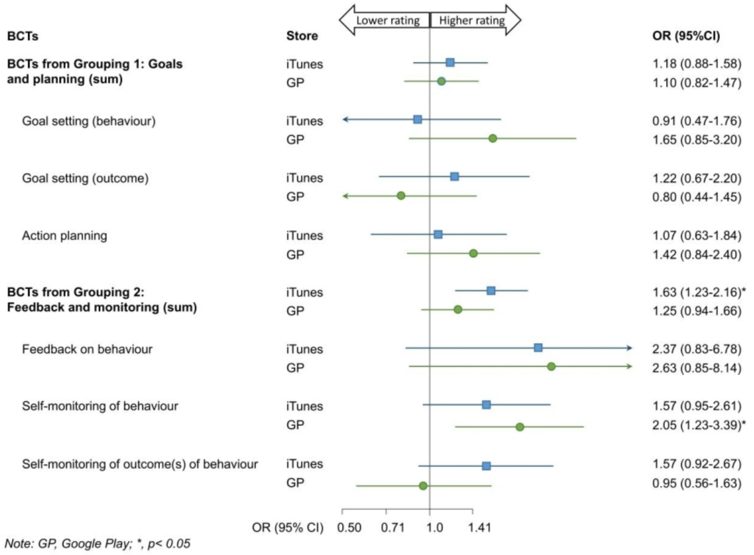

Figure 5: ORs for the associations between BCTs that have been shown to be effective and a 4- or 5- star rating

140x78mm (300 x 300 DPI)

Supplementary file 1: Sensitivity analysis including only those BCTs for which there was high level of confidence in the presence of the BCT (++)

Variable	store	OR (CI)	store	OR (CI)
Group 1: Goals and planning	iTunes	1.16 (0.87-1.55)	GP	1.11 (0.83-1.49)
1.1. Goal setting (behavior)	iTunes	0.91 (0.47-1.76)	GP	1.65 (0.85-3.20)
1.3. Goal setting (outcome)	iTunes	1.18 (0.64-2.18)	GP	0.81 (0.44-1.49)
1.4. Action planning	iTunes	1.07 (0.63-1.84)	GP	1.42 (0.84-2.40)
Group 2: Feedback and monitoring	iTunes	1.62 (1.22-2.13)*	GP	1.25 (0.95-1.64)
2.2. Feedback on behaviour	iTunes	2.07 (1.02-4.22)*	GP	1.76 (0.84-3.68)
2.3. Self-monitoring of behaviour	iTunes	1.57 (0.95-2.61)	GP	2.05 (1.23-3.39)*
2.4. Self-monitoring of outcome(s) of behaviour	iTunes	1.61 (0.93-2.77)	GP	0.98 (0.57-1.69)

Feedback on behavior crosses into significance for iTunes. Otherwise, the results are very consistent with the original findings.

140x78mm (300 x 300 DPI)

Reporting checklist for quality improvement study.

Based on the SQUIRE guidelines.

Instructions to authors

Complete this checklist by entering the page numbers from your manuscript where readers will find each of the items listed below.

Your article may not currently address all the items on the checklist. Please modify your text to include the missing information. If you are certain that an item does not apply, please write "n/a" and provide a short explanation.

Upload your completed checklist as an extra file when you submit to a journal.

In your methods section, say that you used the SQUIRE reporting guidelines, and cite them as:

Ogrinc G, Davies L, Goodman D, Batalden P, Davidoff F, Stevens D. SQUIRE 2.0 (Standards for QUality Improvement Reporting Excellence): revised publication guidelines from a detailed consensus process

For peer review only

		Reporting Item	Page Number
	#1	Indicate that the manuscript concerns an initiative to improve healthcare (broadly defined to include the quality, safety, effectiveness, patientcenteredness, timeliness, cost, efficiency, and equity of healthcare)	2
	#02a	Provide adequate information to aid in searching and indexing	1
	#02b	Summarize all key information from various sections of the text using the abstract format of the intended publication or a structured summary such as: background, local problem, methods, interventions, results, conclusions	1
Problem description	#3	Nature and significance of the local problem	1
Available knowledge	#4	Summary of what is currently known about the problem, including relevant previous studies	1
Rationale	#5	Informal or formal frameworks, models, concepts, and / or theories used to explain the problem, any reasons or assumptions that were used to develop the intervention(s), and reasons why the intervention(s) was	1

		expected to work	
Specific aims	#6	Purpose of the project and of this report	2
Context	#7	Contextual elements considered important at the outset of introducing the intervention(s)	2
Intervention(s)	#08a	Description of the intervention(s) in sufficient detail that others could reproduce it	2
	#08b	Specifics of the team involved in the work	2
Study of the Intervention(s)	#09a	Approach chosen for assessing the impact of the intervention(s)	2
	#09b	Approach used to establish whether the observed outcomes were due to the intervention(s)	See note 1
Measures	#10a	Measures chosen for studying processes and outcomes of the intervention(s), including rationale for choosing them, their operational definitions, and their validity and reliability	3,4
	#10b	Description of the approach to the ongoing assessment of contextual elements that contributed to the success, failure, efficiency, and cost	3,4
	#10c	Methods employed for assessing completeness and accuracy of data	See note 2
Analysis	#11a	Qualitative and quantitative methods used to draw inferences from the data	See note 3
	#11b	Methods for understanding variation within the data, including the effects of time as a variable	4
Ethical considerations	#12	Ethical aspects of implementing and studying the intervention(s) and how they were addressed, including, but not limited to, formal ethics review and potential conflict(s) of interest	See note 4
	#13a	Initial steps of the intervention(s) and their evolution over time (e.g., time-line diagram, flow chart, or table), including modifications made to the intervention during the project	5
	#13b	Details of the process measures and outcome	5
	#13c	Contextual elements that interacted with the intervention(s)	6
	#13d	Observed associations between outcomes, interventions, and relevant	6

For peer review only

		contextual elements	
	#13e	Unintended consequences such as unexpected benefits, problems, failures, or costs associated with the intervention(s).	See note 5
	#13f	Details about missing data	See note 6
Summary	#14a	Key findings, including relevance to the rationale and specific aims	8
	#14b	Particular strengths of the project	8
Interpretation	#15a	Nature of the association between the intervention(s) and the outcomes	8
	#15b	Comparison of results with findings from other publications	8
	#15c	Impact of the project on people and systems	9
	#15d	Reasons for any differences between observed and anticipated outcomes, including the influence of context	8
	#15e	Costs and strategic trade-offs, including opportunity costs	9
Limitations	#16a	Limits to the generalizability of the work	8,9
	#16b	Factors that might have limited internal validity such as confounding, bias, or imprecision in the design, methods, measurement, or analysis	8,9
	#16c	Efforts made to minimize and adjust for limitations	8,9
Conclusion	#17a	Usefulness of the work	9
	#17b	Sustainability	See note 7
	#17c	Potential for spread to other contexts	9
	#17d	Implications for practice and for further study in the field	9
	#17e	Suggested next steps	9
Funding	#18	Sources of funding that supported this work. Role, if any, of the funding organization in the design, implementation, interpretation, and reporting	See note 8

Author notes

1. 9 - this is considered in the limitations of the approach

2. 3: two researchers extracted the data

For peer review only

3. 4: quantitative methods used as this was appropriate for the research methods used

4. N/A as data was derived from the app stores

5. N/A for this study as the data was derived from the app stores hnce we worked within the limits of the data available to us.

6. N/A as no missing data

7. N/A because of the nature of the study using data derived from app stores

8. N/A as separate sheet as instructed in the submission

The SQUIRE 2.0 checklist is distributed under the terms of the Creative Commons Attribution License CC BY-NC 4.0. This checklist was completed on 23. October 2018 using http://www.goodreports.org/, a tool made by the EQUATOR Network in collaboration with Penelope.ai

For peer review only

BMJ Open

Relationship between popularity and the likely efficacy: an observational study based on a random selection on top-ranked physical activity apps

Journal:	*BMJ Open*
Manuscript ID	bmjopen-2018-027536.R1
Article Type:	Original research
Date Submitted by the Author:	01-Jul-2019
Complete List of Authors:	Bondaronek, Paulina; University College London, eHealth Unit, Research Department of Primary Care and Population Health<br>Slee, April; University College London, Research Department of Primary Care and Population Health<br>Hamilton, Fiona; University College London, eHealth Unit, Research Department of Primary Care and Population Health<br>Murray, Elizabeth; University College London, Primary Care and Population Health
**Primary Subject Heading**:	Public health
Secondary Subject Heading:	Health services research, Health policy, Sports and exercise medicine
Keywords:	mHealth, PUBLIC HEALTH, Physical Activity, Behaviour Change, Intervention

SCHOLARONE™
Manuscripts

Relationship between popularity and the likely efficacy: an observational study based on a random selection on top-ranked physical activity apps

Bondaronek Paulina [1], Slee April [2], Hamilton Fiona [1], Murray Elizabeth [1]

1 - eHealth Unit, Research Department of Primary Care and Population Health, University College London

2 - Research Department of Primary Care and Population Health, University College London

Corresponding author: Paulina Bondaronek, eHealth Unit, Department of Primary Care & Population Health, University College London, Upper Third Floor, UCL Medical School (Royal Free Campus) Rowland Hill Street, London NW3 2PF; email: p.bondaronek@ucl.ac.uk; phone: 07742966769

Word count: 4432

For peer review only

ABSTRACT

Objectives: To explore the relationship between popularity of mobile application (apps) for physical activity (PA) and their likely efficacy. The primary objective was to assess the association between app popularity (indicated by user ratings) and likely efficacy (indicated by the number of Behaviour Change Techniques, BCTs, present). The secondary objective was to assess the relationship between user ratings and those BCTs that have been shown to be effective in increasing PA.

Design: Observational study

Methods: 400 top-ranked free and paid apps from iTunes and Google Play stores were screened, and were included if the primary behaviour targeted was PA and they had stand-alone functionality. The outcome variable of user rating was dichotomised into high (4,5 stars) or low (1,2,3 stars) ratings.

Setting: iTunes and Google Play app stores

Participants: No individual participants but the study utilised use-led rating system in the app store
Interventions: Popular publicly available PA apps

Primary and secondary outcome measures: BCTs and user rating

Results: Of 400 apps, 156 were eligible and 65 apps were randomly selected, downloaded and assessed by two reviewers. There was no relationship overall between star ratings and the number of BCTs present, nor between star ratings and the presence of BCTs known to be effective in increasing PA. App store was strongly associated with star ratings, with lower likelihood of finding 4 or 5 stars in iTunes compared to Google Play (Odds ratio, OR: 0.74 95% CI: 0.73-0.76, p< 0.001).

Conclusions: The findings of this study suggest that popularity does not necessarily imply the likelihood of effectiveness. Hence, public health impact is unlikely to be achieved by allowing market forces to “prescribe” what is used by the public.

For peer review only - http://bmjopen.bmj.com/site/about/guidelines.xhtml

Strengths and limitations of this study

- First study to assess the relationship between popularity of physical activity apps and their likely efficacy in two major app stores
- A systematic approach to sample identification and assessment including a standardised assessment tool used to characterise behaviour change interventions
- Sample identified and assessed by two independent reviewers
- It is unknown what variables comprise the ranking algorithm from which the sample was derived
- It is possible that user ratings, as expressed by the stars assigned to the apps, can relate to different aspects of the app content and functionalities

Keywords: mHealth, PUBLIC HEALTH, Physical Activity, Behaviour Change, Intervention

For peer review only

INTRODUCTION

The accessibility, convenience, and wide reach of apps create new avenues for health behaviour change on a large scale. Out of the total 325,000 health apps available on the market in 2017 [1] the largest app groups were fitness apps. The rapidly increased market supply of the apps reflects public demand for the new means of engaging in health behaviours. In 2015, 34% percent of mobile phone owners had at least one health-related app downloaded on their mobile phone [2]. Yet, 28.7% of adults in England are inactive [3]. This discrepancy may be explained by the difference between the intention to increase PA and the actual engagement in the behaviour, i.e, the intention-behaviour gap[4].

Researchers assessing the relationship between the popularity of apps and their quality has found mixed results. Azar, et al. [5] found that apps for weight management that were of higher quality, defined in the their study as inclusion of the constructs from four behaviour change theories, were not among the highest ranked apps the app stores. Similarly, apps that had higher download rates or higher ranking were associated with less adherence to guidelines in smoking cessation apps [6]. On the other hand, Pereira-Azevedo, et al. [7] reviewed the descriptions of 129 urology apps in the Google Play app store and found that higher download rates were associated with expert involvement in the development of the apps. These studies targeted different health conditions and behaviours from physical activity, the subject of this study, and used different definitions of quality, such as consistency with behaviour change theory, expert involvement, and adherence to guidelines, hence their findings need to be interpreted with caution. None of these studies used behaviour change theory to systematically assess the content of the apps in terms of their likely efficacy and how it relates to app popularity.

The Behaviour Change Technique (BCT) Taxonomy [8] is a systematic method used to specify the content of behaviour change interventions [9]. The Taxonomy has also been used to quantify the inclusion of behaviour change theory in interventions, including apps [10-12]. For example, Bardus, et al. [10] extracted BCTs from 23 apps aimed at weight management. They used the Mobile App Rating Scale (MARS) to assess the quality of apps [13] and showed a positive association between the number of BCTs and the MARS subsets: Engagement, Functionality, and Aesthetics, as well as the overall quality of app score. This may suggest that the inclusion of BCTs may be related to the quality of the apps, as assessed by MARS.

However, the same authors found no association between user ratings and either BCTs or the quality indicators on the MARS scale suggesting that apps that were highly rated were not of high quality. Similarly, Crane, et al. [11] showed that only 1 BCT, “prompt review of goals”, was associated with user ratings in alcohol reduction apps. The authors concluded that there was little association between the mention of theory, BCTs inclusion and popularity of the alcohol reduction apps. These findings indicate that further work is needed to explore whether popular apps are those that are of high quality and are likely to be effective. This has relevance for public health policy as the combination of popularity with likely efficacy would suggest that apps have a potential role in promoting public health, whereas a disconnect between popularity and likelihood of efficacy would raise questions about leaving the market to guide user choices in app downloads.

Systematic reviews and meta-analyses have shown that self-regulation techniques, such as goal setting, monitoring, and feedback are effective in increasing PA [14-17] behaviour and they may have cumulative effects. For example, Michie, et al. [15] showed that self-monitoring with other self-regulatory BCTs were more effective in increasing PA than using one of those BCTs in isolation. Self-regulation has been acknowledged as important constructs in behaviour change theories, e.g. in Control Theory [18] and Self-Regulation Theory [19]. Hence, the presence of these BCTs can be used as an indicator for quality of those apps and a proxy measure of their likely efficacy.

For peer review only - http://bmjopen.bmj.com/site/about/guidelines.xhtml

Aim and objectives

The aim of this study was to explore the relationship between the user ratings as a marker of popularity of apps and the inclusion of BCTs as a marker of likely efficacy in publicly available PA apps. Specifically, the primary objective was to assess the association between user ratings and the number of BCTs included in the sample of popular PA apps. The secondary objective assessed the inclusion of BCTs shown to be effective in increasing PA behaviour, in particular BCTs related to self-regulation of behaviour.

METHODS

Design

The study used a random sample of popular apps to determine the association between user ratings and the presence of BCTs. Descriptive data included the cost and size of the app; the number and distribution of star ratings; and the presence of BCTs.

Data sources and collection

The sample included popular PA apps identified from 400 top-ranked free and paid apps from the Health and Fitness category (100 iTunes free + 100 iTunes paid + 100 Google Play free + 100 Google Play paid) available in the UK version of iTunes and Google Play app stores on 17/10/16. Apps were included if (i) the primary behaviour targeted was PA; (ii) they had stand-alone functionality. Apps that that specifically targeted children were excluded as it is likely that children might not have access to iTunes or Google Play accounts and the ratings might reflect the parental ratings rather than the children. Two reviewers (PB, GA) reviewed each app, assessed whether specific BCTs were present, and extracted relevant data. The detailed process of app identification and sample description is provided in Bondaronek, et al. [20]. Based on the number of ratings, the proportion, and the distribution of star ratings we were able to reconstitute individual level response data within each store.

Data extraction

BCTs extraction as a measure of likely efficacy

The presence or absence of 93 BCTs in the BCT Taxonomy v1 as described by Michie, et al. [8] were classified using 3 categories: absent, appears to be present but evidence is not clear (+), present beyond doubt and evidence clear (++). The presence of self-regulation BCTs, associated with PA intervention effectiveness [14-17] [16]was classified using the BCT Taxonomy Grouping 1: Goal and planning, and Grouping 2: Feedback and monitoring as these groups reflect the self-regulation BCTs in the Taxonomy.

User ratings

We extracted the star ratings (1-5 stars) assigned to the apps in both stores and the number of ratings assigned to each app. The average star rating was calculated by summing the number of stars awarded across all users and dividing them by the number of users that submitted the rating. When an app appeared in both stores, a weighted average of the star ratings for each store was calculated based on the relative proportion of the ratings in each store. This algorithm is equivalent to summing the number of stars awarded by all users in both stores and then dividing them by the total number of reviews across both stores. This calculation weights users equally regardless of the platform used to access the app. The variable was dichotomised into high (4,5 stars) or low (1,2,3 stars) ratings.

Variables determined a priori potentially to affect the relationship between BCTs and higher or lower ratings were app store (iTunes or Google Play), number of features, whether the app was free or required payment, size (in megabytes, MB) and usability.

For peer review only - http://bmjopen.bmj.com/site/about/guidelines.xhtml

Number of features

Health apps use technology-enhanced features to deliver BCTs in order to influence behaviour. The apps were categorised according to the features offered by the app, e.g., PA tracking, reminders, app community, data visualisation, etc.). To the authors' best knowledge, at the time of writing, there was no standardised list of features that are commonly used in PA apps. The first ten apps extracted were used to compile a list of PA app features. The list was continuously updated throughout the app extraction process in order to accommodate for new features that were found in the PA app sample. The features were extracted by two reviewers (PB, GA and any discrepancies were resolved by comparing the results of the extraction and reaching consensus).

Usability

Usability was assessed using the System Usability Scale, SUS, [21] which consists of 10 items ranked on a 5-point Likert scale, from "strongly disagree" to "strongly agree". To make the wording of the scale more applicable to the study, two changes were made: 1) the wording of the 8th statement was changed from "cumbersome" to "awkward" as recommended [22-24]; 2) the word "system" was replaced by "app". SUS yields scores from 0-100 where higher scores indicate greater usability. The interpretation of the SUS score used the thresholds proposed and validated by Bangor, et al. [22] with 72.5 described as good usability.

Data analysis

Agreement statistics including the prevalence and bias adjusted kappa statistic, PABAK [25] and unadjusted kappa [26] were calculated. Disagreements were then resolved by discussions among the two reviewers and consultation with other authors if unresolved.

The number of BCTs in the apps was summarized using the mean, standard deviation, median, 25th and 75th percentiles, and the maximum and minimum. Similar statistics were used to summarize user ratings, cost, size, and SUS score. The summary descriptive tables were presented for free and paid apps separately and in total as app stores have separate rankings based on the cost.

The pre-specified primary analysis was based on a linear regression of the number of BCTs on star average as this continuous outcome required the smallest sample of apps for adequate statistical power. Logistic regression to determine the relationship between the number of BCTs and the odds of high and low star rating was the pre-specified secondary analysis. We modelled a higher (versus lower) star rating as the event.

Sample size calculations

The sample size calculation was based on pilot sample of 10 apps (5 paid from iTunes, 5 free from Google Play) selected from the 400 app identified. Apps were sorted in order of store rankings. From iTunes, 38 potentially eligible paid apps were identified and every 8th app was included in the pilot sample (N = 38/5 =7.6= ~8). From Google Play, 55 potentially eligible free apps were identified and every 11th app was included in the pilot sample (N = 55/5 =11). If a sample app was downloaded and found to be ineligible, the next lowest ranked app was used instead. Three apps from Google Play and none from iTunes were found to be ineligible and replaced.

A pilot study based on the 10 apps suggested that mean star ratings were normally distributed. Hence, a sample size calculation was undertaken using mean ratings as a continuous measure in a linear model. Based on this framework, a sample size of 51 apps would provide 90% power to detect a change of 0.11 for each additional BCT at 5% significance level (type I error rate). A sample size of 65 apps was selected to account for any randomly selected apps that might not fulfil the inclusion criteria once downloaded. However, the pilot sample was not sufficiently large enough to show that there was a skew in star average for the complete sample once data were extracted, which would preclude the use of linear models. The difference in star rating by store was also unknown at the time of study planning, and hence the app store interactions were a post-hoc addition to this plan. A retrospective power analysis was conducted. Based on simulations, this study had 97% power to detect an OR >= 1.2 with 64 apps. Since we had high power and did not see a significant result, this suggests that the true increase in the chance of a star rating of 4 or 5 with each additional BCT has an OR of < 1.2.

For peer review only - http://bmjopen.bmj.com/site/about/guidelines.xhtml

As indicated in the analysis plan, star ratings of 4 and 5 were classified as high ratings, while ratings of 1, 2 and 3 were classified as low ratings. We used the user ratings data from each store in regression models with a random effect to account for store differences. Modelling proceeded in a series of steps. First, we tested a small set of variables identified in the analysis plan for the relationship to star rating (cost, usability, number of features, size) by including each of these as fixed covariates in univariate logistic regression models with the outcome of high versus low ratings. Any variables significant at $p \leq 0.1$ were included in models examining BCTs. App store and the app store-by-BCT interaction terms were included in each model to reflect that the clientele for iTunes and Google Play may be different, and that these differences may impact the relationship between BCTs and star ratings. Weighted logistic regression (by the number of responses for each app) was used with a random intercept term for app, reflecting that the apps selected for analysis were a random sample of available PA apps, and to control for correlation when the same app was reviewed in both stores. Analyses were performed using SAS version 9.3 and R version 3.3.3.

Sensitivity analysis

Sensitivity analyses included a revision of the number of BCTs including only those that were present beyond any doubt (++) [27], and dichotomising user ratings into 5 stars versus < 5 stars.

Patient and Public Involvement

This research was conducted without patient involvement.

RESULTS

Sample

Out of 400 apps, 244 apps were excluded (see Figure 1). Of the remaining 156 PA apps, 31 were duplicates in that the same app appeared in both iTunes and Google Play. Thus, 125 unique apps were eligible for random selection; 65 unique apps were randomly selected for analysis including 32 free apps and 33 paid apps. One app, Break, was excluded from analyses of user reviews as the data on user ratings were not available due to the small number of ratings, resulting in a final sample of 64 PA apps. These apps collectively received more than 2.8 million user ratings.

Figure 1: Flow chart of the apps included in the analysis

<Figure 1 separate file>

There was substantial agreement between the reviewers in the assessment of BCTs (PABAK =0.94, 95% CI 0.93 to 0.95, Kappa =0.78, 95% CI 0.75 to 0.81).

Characteristics of the apps are displayed in Table 1. The number of BCTs and features were approximately normally distributed, with the mean number of BCTs being 7.1 (SD= 3.0) and the mean number of features being 5.8 (SD=2.1) (see Supplementary file 1 for list of PA app features and frequency of occurrence). Star ratings, size, SUS score and the cost variables were skewed. The median star rating was 4.5 (IQR 1.9 – 4.9) and the median SUS score was 86.3 (IQR 75.00 – 91.88). Among paid apps, the median cost was £2.40 (IQR 1.78 – 2.99).

Table 1: Characteristics of PA apps included in the sample

For peer review only - http://bmjopen.bmj.com/site/about/guidelines.xhtml

		Free (n=32)	Paid (n=32)	Total (n=64)
Number of BCTs	Mean ± SD	6.56 ± 2.99	7.56 ± 2.87	7.06 ± 2.95
	Median	7.00	8.00	8.00
	25, 75%tile	5.00, 8.00	6.00, 10.00	5.00, 9.00
Star average*	Mean ± SD	4.26 ± 0.65	4.35 ± 0.45	4.30 ± 0.56
	Median	4.50	4.49	4.50
	25, 75%tile	4.30, 4.60	4.29, 4.60	4.30, 4.60
Number of Features	Mean ± SD	5.94 ± 2.17	5.72 ± 1.95	5.8 ± 2.05
	Median	7.00	8.00	6.00
	25, 75%tile	4.00, 8.00	5.00, 8.00	4.00, 8.00
Size (MB)*	Mean ± SD	45.53 ± 40.85	61.51 ± 47.22	53.52 ± 44.53
	Median	32.45	53.97	39.64
	25, 75%tile	16.82, 62.27	28.92, 85.02	25.52, 79.50
Usability (SUS)	Mean ± SD	81.25 ± 12.64	85.23 ± 12.02	83.24 ± 12.40
	Median	85.00	87.50	86.25
	25, 75%tile	71.88, 91.25	78.75, 93.75	75.00, 91.88
Cost (GBP) *	Mean ± SD		3.00 ± 2.12	
	Median	N/A	2.35	N/A
	25, 75%tile		1.78, 2.99	

*weighted average of iTunes and Google Play ; GBP: British pounds, N/A, not applicable

User ratings

In total, 2,819,469 user ratings of the 64 apps were used in the analysis. Among these, 88.5% were 4 or 5 star reviews and 11.5% were 1, 2 or 3-star reviews. Among the covariates considered for model inclusion, good usability increased the chance of a 4 or 5 star rating compared to poor usability (OR: 1.66, 95% CI 0.96 to 2.89, p= 0.071). This covariate was included in all subsequent models. App store was strongly associated with star ratings, with lower likelihood of finding 4 or 5 stars in iTunes compared to Google Play (OR: 0.74 95% CI: 0.73 to 0.76, p< 0.001).

Further investigation of the difference between the stores and user ratings showed that there was 37% higher likelihood of awarding 4 or 5 stars in Google Play compared to iTunes (95%CI 35% to 41%, p < 0.001). A similar pattern can be seen comparing the weighted average within-app star rating across stores. There were 17 apps that were sold in both stores (Figure 2). Analysis was conducted to assess whether the proportion with the high star ratings in Google Play exceeds what would have been found by chance. If there were no relationship between store and star rating, we would expect the star rating in iTunes to exceed the star rating in Google Play for about 50% of apps (coin flip). Mean star rating in Google Play exceeded the rating in iTunes in 13/17 apps (76.5%, 95% CI: 64.2 to 86.2, p<0.0001). Since

For peer review only - http://bmjopen.bmj.com/site/about/guidelines.xhtml

the analysis was significant, we can rule out that the difference between the user ratings in the stores was due to chance. Cost, size and number of features were not related to star ratings.

Figure 2: The comparison of star average between the same apps that existed in both stores (n=17)

<Figure 2 separate file>

Primary analysis

The primary analysis of the association between user ratings and the overall number of BCTs included in each app found no relationship between number of BCTs and star rating (OR: 1.05, 95% CI: 0.97 to 1.14, p =0.236). Subgroup analysis showed that in iTunes only, a higher star rating was associated with the total number of BCTs (OR: 0.72, 95% CI: 0.71 to 0.74, p< 0.001).

Based on the model containing the store interaction (Figure 3), in iTunes there was an association with each additional BCT corresponding to 15% increase in the likelihood of a higher star rating (OR: 1.15, 95% CI: 1.06, 1.25), there was no association between the number of BCTs and Google Play. Usability was not significant in the multivariate model (OR 1.57, 95% CI: 0.91, 2.71)

Figure 3: ORs for the associations between the number of BCTs and a 4- or 5- star rating

<Figure 3 separate file>

Secondary objective

The secondary analysis examined the associations between a 4- or 5- star user rating and the inclusion of BCTs that have been shown to be effective in increasing PA behaviour.

The frequency of the BCTs associated with efficacy for increasing PA behaviour is presented in Figure 4.
The BCT grouping 1 (Goal and planning) was represented in 84.4% of evaluated apps. The most common of these was goal setting for behaviours (84.4%), followed by action planning (35.9%) and goal setting for outcomes (18.8%). The BCT grouping 2 (Feedback and monitoring) was included in 92.2% of evaluated apps. Feedback on behaviour was included in nearly all apps (90.6%), while self-monitoring of behaviour and of outcomes were incorporated in about a third of apps.

Figure 4: Frequency of individual BCTs within the two Groupings of self-regulatory BCTs

<Figure 4 separate file>

The number of Grouping 1 BCTs had no impact on star rating in either store (Figure 5). Among iTunes users, each additional BCT from Grouping 2 increased the chance of a high star rating by 63% (OR: 1.63 95% CI: 1.23-2.16*).* There was no association between the number of BCTs and star rating among Google Play users. Self-monitoring of behaviour was associated with higher ratings in Google Play (OR: 2.05, 95% CI: 1.23-3.39)

Figure 5: ORs for the associations between BCTs that have been shown to be effective and a 4- or 5- star rating
<Figure 5 separate file>

For peer review only - http://bmjopen.bmj.com/site/about/guidelines.xhtml

For peer review only

Sensitivity analysis

The sensitivity analyses including only those BCTs that were classified as "present beyond all reasonable doubt" [27] showed that for iTunes, the BCT 2.2 Feedback on behaviour crossed into significance. Otherwise, the results are consistent with the original findings. The results of the sensitivity analysis are available in the Supplementary file 2.

DISCUSSION

The aim of this study was to assess the relationship between the popularity of publicly available PA apps (assessed through user ratings) and their likely efficacy (assessed through the inclusion of BCTs). Overall, for both app stores, there was no association between popularity and likely efficacy as indicated by the overall presence of BCTs and the BCTs known to be effective in increasing PA. However, users in each app stores differed and there was an association between the number of BCTs and high user rating in iTunes but not in Google Play.

This study supports the findings of previous research in apps targeting weight management [5 10], smoking cessation [6] and alcohol reduction [11] which showed that apps that were highly rated, highly ranked or highly downloaded were not necessarily of high quality. No association between the BCTs and user ratings suggests other factors may be contributing to the apps popularity. High quality graphic design, visual appeal and ease of use are more likely to attract potential customers to download and engage with the app. In addition, the promotion of the apps can play a role in the download rates.

The finding of an association between user rankings and overall number of BCTs in one store (iTunes) but not the other (Google Play) is new. There are various possible explanations for this finding. Market researchers suggest possible differences in the population of users of each store [28 29]. For example, iPhone users might be more affluent, they engage with their device for longer, make more purchases with their phone, and are more loyal to their brand [30]. In addition, there are differences in the review and approval processes between the two stores which could have influence the user ratings [31]. Future studies should explore this observation further, and determine whether it holds for health apps targeting different behaviours.

Strengths

The main strength of the study includes the systematic assessment of the app content conducted by two reviewers. The sample was identified from the most popular publicly available apps from two major app distribution platforms. The use of BCT taxonomy provides a standardised assessment tool, which has been utilised in other studies assessing the content of apps [12 32-34]. Second, Guzman [35] argued that the star rating represents an average score for the whole app that combines both positive and negative evaluations aggregated across users. The study, however, used rater-level data which included individual ratings from 2.8 million users. These large numbers mitigate the problem posed by averaging the star rating across users.

Limitations

This study has some important limitations. First, the main limitation of the study relates to the variables used in this study. It is possible that user ratings, as expressed by the stars assigned to the apps, can relate to different aspects of the app functioning and content. There is evidence suggesting that app reviews tend to occur near a new release which can suggest that the ratings may include comments on the specific updates of the software [36]. In addition, the possibility that user ratings were influenced by fake reviews cannot be excluded [37 38]. However, the user rating was considered the most appropriate measure to explore since it represents a user-led feedback that reflects user experience. Similarly, the choice of the BCTs as an approximation for likely efficacy was selected because studies assessing the efficacy of the apps on the market are scarce [39-41]. Second, the ranking algorithms from which the sample was derived is unknown. Hence, this lack of transparency prevented evaluation of how the calculation of rank might

For peer review only - http://bmjopen.bmj.com/site/about/guidelines.xhtml

have influenced the app selection. However, apps appear in rank order by default in app stores hence the rank affects what users are seeing. As the aim of the study was to assess the most popular apps, the choice of highly ranked apps was considered the most appropriate for the context of this research. Third, Google Play market tend to have more ratings than iTunes because the process of app review is more complex in the latter [42]. This was addressed in the study by using the weighted averages of the ratings across the stores (for the summary of the app characteristics), and by controlling for store in the regression models. While there is a difference in the app review process in both app stores, feedback from both stores should be recognised as valid and important. Fourth, while we inspected the apps to ensure that the duplicate apps were similar in both stores, there could be differences in the functionality of the apps between the app stores that we could not see. Finally, failure to detect the skew in the original primary outcome used to power the study is a limitation. However, a retrospective power analysis showed we had high power to detect an OR of 1.2 and did not find significant result, hence the true OR is likely to be < 1.2.

Implications

In this study, we showed no evidence of association between popularity and likely efficacy. Hence, we suggest that, at present, allowing the commercial market to determine which PA apps are downloaded is unlikely to be an effective method of public health promotion in terms of increasing overall levels of PA.

Further research is needed to understand which BCTs, and in which combination, are most effective in increasing PA when delivered via an app; how these BCTs can best be delivered; and how to combine features which promote efficacy with those that promote popularity. Last, the differences in iTunes and Google Play users is an unexpected finding, and not one that the study set out to identify, (i.e. not an a priori hypothesis). Future researchers should be aware of the potential differences between iTunes and Google Play users and ensure research is carried out on both platforms.

Based on the findings, we suggest some implications for public health policy. Apps aimed to increase PA represent the largest category in the two major app stores [43] which illustrates public demand for engaging in PA. The lack of quality in those apps indicates a missed opportunity to increase health at the population level. Initiatives to identify and promote high quality apps are in development, e.g., the NHS Apps Library [44]. However, there is an urgent need to evaluate the effectiveness of these apps with potential users.

Conclusion

To date, this is the first study to assess the association between popularity (measured using user ratings of the apps) and likely efficacy (measured using the inclusion of the BCTs) of publicly available highly-ranked PA apps available in the major app stores. No relationship was found between popularity and likely efficacy suggesting that popularity does not assure high quality, and what is liked may not be what is likely to be effective. However, PA apps in this study were highly rated, highly-ranked apps from the major app stores, hence highly visible for the potential user. Hence, promotion of public health is unlikely to be achieved by allowing market forces to determine which PA apps are used. More studies are needed to assess the effectiveness of apps with users in a real-world setting to investigate the app components that are both, effective and valued by the users.

Supplementary files

Supplementary file 1: Definition of PA app features and the frequency of occurrence
Supplementary file 2: Sensitivity analysis including BCTs with only high level of confidence in the presence in the apps (++)

Competing Interests
None declared.

For peer review only - http://bmjopen.bmj.com/site/about/guidelines.xhtml

Data Sharing Statement

All data used in the study is available upon request.

Contributorship statement

PB designed the study, collected the data, analysed the data and wrote the manuscript. AS analysed the data and co-wrote the manuscript. EM and FH provided advice on study design, data analysis and interpretation, and contributed to the critical appraisal of the manuscript.

Acknowledgements

We wish to acknowledge Ghadah Alkhaldi (Community Health Sciences Department, College of Applied Medical Sciences, King Saud University, Riyadh, Saudi Arabia) for her help with the data extraction.

Funding

PB is a PhD student funded by the Medical Research Council.

References

1. research2guidance. mHealth App Economics: Current Statu and Future Trends in Mobile Health: research2guidance; 2017 [Available from: https://www.uzelf.org/wp-content/uploads/2017/12/R2G-mHealth-Developer-Economics-2017-Status-And-Trends.pdf accessed 20/09/18 2018.
2. Terry K. Number of health apps soars, but use does not always follow 2015 [Available from: http://www.medscape.com/viewarticle/851226.
3. Public Health England. Public Health Outcomes Framework: Physical Activity 2016 [Available from: http://www.phoutcomes.info/search/physical%20activity#pat/6/ati/102/par/E12000004.
4. Rhodes RE, Dickau L. Moderators of the intention-behaviour relationship in the physical activity domain: a systematic review. *Br J Sports Med* 2013;47(4):215-25.
5. Azar KM, Lesser LI, Laing BY, et al. Mobile applications for weight management: theory-based content analysis. *Am J Prev Med* 2013;45(5):583-9. doi: 10.1016/j.amepre.2013.07.005
6. Abroms LC, Padmanabhan N, Thaweethai L, et al. iPhone apps for smoking cessation: a content analysis. *American journal of preventive medicine* 2011;40(3):279-85.
7. Pereira-Azevedo N, Osório L, Cavadas V, et al. Expert involvement predicts mHealth app downloads: multivariate regression analysis of urology apps. *JMIR mHealth and uHealth* 2016;4(3) doi: 10.2196/mhealth.5738
8. Michie S, Richardson M, Johnston M, et al. The behavior change technique taxonomy (v1) of 93 hierarchically clustered techniques: building an international consensus for the reporting of behavior change interventions. *Annals of behavioral medicine* 2013;46(1):81-95. doi: 10.1007/s12160-013-9486-6
9. Michie S, Atkins L, West R. The behaviour change wheel. *A guide to designing interventions 1st ed Great Britain: Silverback Publishing* 2014
10. Bardus M, van Beurden SB, Smith JR, et al. A review and content analysis of engagement, functionality, aesthetics, information quality, and change techniques in the most popular commercial apps for weight management. *Int J Behav Nutr Phys Act* 2016;13:35. doi: 10.1186/s12966-016-0359-9
11. Crane D, Garnett C, Brown J, et al. Behavior change techniques in popular alcohol reduction apps: content analysis. *J Med Internet Res* 2015;17(5):e118. doi: 10.2196/jmir.4060
12. Middelweerd A, Mollee JS, van der Wal CN, et al. Apps to promote physical activity among adults: a review and content analysis. *Int J Behav Nutr Phys Act* 2014;11(1):97.

For peer review only - http://bmjopen.bmj.com/site/about/guidelines.xhtml

13. Stoyanov SR, Hides L, Kavanagh DJ, et al. Mobile app rating scale: a new tool for assessing the quality of health mobile apps. *JMIR Mhealth Uhealth* 2015;3(1):e27. doi: 10.2196/mhealth.3422
14. Greaves CJ, Sheppard KE, Abraham C, et al. Systematic review of reviews of intervention components associated with increased effectiveness in dietary and physical activity interventions. *BMC public health* 2011;11(1):119.
15. Michie S, Abraham C, Whittington C, et al. Effective techniques in healthy eating and physical activity interventions: a meta-regression. *Health Psychology* 2009;28(6):690. doi: 10.1037/a0016136
16. Conn VS, Hafdahl AR, Mehr DR. Interventions to increase physical activity among healthy adults: meta-analysis of outcomes. *American journal of public health* 2011;101(4):751-58.
17. Pearson ES. Goal setting as a health behavior change strategy in overweight and obese adults: a systematic literature review examining intervention components. *Patient education and counseling* 2012;87(1):32-42. doi: 10.1016/j.pec.2011.07.018
18. Carver CS, Scheier MF. Control theory: A useful conceptual framework for personality–social, clinical, and health psychology. *Psychological bulletin* 1982;92(1):111.
19. Kanfer FH. Implications of a self-regulation model of therapy for treatment of addictive behaviors. Treating addictive behaviors: Springer 1986:29-47.
20. The public health potential of the current health apps for increasing physical activity. Proceedings of the 2017 International Conference on Digital Health; 2017. ACM.
21. Brooke J. SUS-A quick and dirty usability scale. *Usability evaluation in industry* 1996;189(194):4-7.
22. Bangor A, Kortum PT, Miller JT. An empirical evaluation of the system usability scale. *Intl Journal of Human–Computer Interaction* 2008;24(6):574-94. doi: 10.1080/10447310802205776
23. Finstad K. The system usability scale and non-native english speakers. *Journal of usability studies* 2006;1(4):185-88.
24. Lewis JR. Psychometric evaluation of the PSSUQ using data from five years of usability studies. *International Journal of Human-Computer Interaction* 2002;14(3-4):463-88. doi: 10.1080/10447318.2002.9669130
25. Byrt T, Bishop J, Carlin JB. Bias, prevalence and kappa. *Journal of clinical epidemiology* 1993;46(5):423-29. doi: 10.1016/0895-4356(93)90018-V
26. Cohen J. Kappa: Coefficient of concordance. *Educ Psych Measurement* 1960;20(37)
27. Wood CE, Richardson M, Johnston M, et al. Applying the behaviour change technique (BCT) taxonomy v1: a study of coder training. *Translational behavioral medicine* 2014;5(2):134-48.
28. Götz FM, Stieger S, Reips U-D. Users of the main smartphone operating systems (iOS, Android) differ only little in personality. *PloS one* 2017;12(5):e0176921.
29. Monetate. Ecommerce Quarterly 2016: Mobile ate the world and is still hungry 2017 [Available from: http://info.monetate.com/rs/092-TQN-434/images/Monetate-EQ3-2016.pdf?mkt_tok=eyJpIjoiTURFMFptTTNNR1prWWpRMyIsInQiOiJsaDE4bXF1OTA4R0o4T0xjNGZjXC9JRkcxcHM4WkRTZFZkT2tnWWorMGtIc24zSUFsanN2Z2tOVDJHTDZFc3F1eTZnZEZJaWJsQUxzQWRjN3F6ZXVNc2wrcVREMDFJc3RMQU5yc0NGdXU4OG41Zk9Gdk1OaGZRUXNEcisreWFEczcifQ%3D%3D accessed 21/09/18 2018.
30. Katariya J. Apple Vs Android — A comparative study 2017: AndroidPub; 2017 [Available from: https://android.jlelse.eu/apple-vs-android-a-comparative-study-2017-c5799a0a1683 accessed 2018 2017.
31. Why Do Users Install and Delete Apps? A Survey Study. International Conference of Software Business; 2017. Springer.
32. Direito A, Dale LP, Shields E, et al. Do physical activity and dietary smartphone applications incorporate evidence-based behaviour change techniques? *BMC Public Health* 2014;14(1):1.
33. Chen J, Cade JE, Allman-Farinelli M. The Most Popular Smartphone Apps for Weight Loss: A Quality Assessment. *JMIR Mhealth Uhealth* 2015;3(4):e104. doi: 10.2196/mhealth.4334
34. Yang CH, Maher JP, Conroy DE. Implementation of behavior change techniques in mobile applications for physical activity. *Am J Prev Med* 2015;48(4):452-5. doi: 10.1016/j.amepre.2014.10.010

For peer review only - http://bmjopen.bmj.com/site/about/guidelines.xhtml

35. How do users like this feature? a fine grained sentiment analysis of app reviews. Requirements Engineering Conference (RE), 2014 IEEE 22nd International; 2014. IEEE.
36. User feedback in the appstore: An empirical study. Requirements Engineering Conference (RE), 2013 21st IEEE International; 2013. IEEE.
37. AppAware: which mobile applications are hot? Proceedings of the 12th international conference on Human computer interaction with mobile devices and services; 2010. ACM.
38. Trusting smartphone apps? To install or not to install, that is the question. Cognitive Methods in Situation Awareness and Decision Support (CogSIMA), 2013 IEEE International Multi-Disciplinary Conference on; 2013. IEEE.
39. Direito A, Carraça E, Rawstorn J, et al. mHealth technologies to influence physical activity and sedentary behaviors: Behavior change techniques, systematic review and meta-analysis of randomized controlled trials. *Annals of behavioral medicine* 2017;51(2):226-39.
40. Buechi R, Faes L, Bachmann LM, et al. Evidence assessing the diagnostic performance of medical smartphone apps: a systematic review and exploratory meta-analysis. *BMJ open* 2017;7(12):e018280.
41. Jake-Schoffman DE, Silfee VJ, Waring ME, et al. Methods for evaluating the content, usability, and efficacy of commercial mobile health apps. *JMIR mHealth and uHealth* 2017;5(12)
42. Xu W, Liu Y. mHealthApps: a repository and database of mobile health apps. *JMIR mHealth and uHealth* 2015;3(1):e28. doi: 10.2196/mhealth.4026
43. research2guidance. mHealth app developer economics. The state of the art of mHealth application publishing: research2guidance 2014 [updated 5 May 2016. Available from: http://research2guidance.com/product/mhealth-app-developer-economics-2014/.
44. NHS. NHS Apps Library: NHS; [Available from: https://www.nhs.uk/apps-library/ accessed 16/06/19.

For peer review only

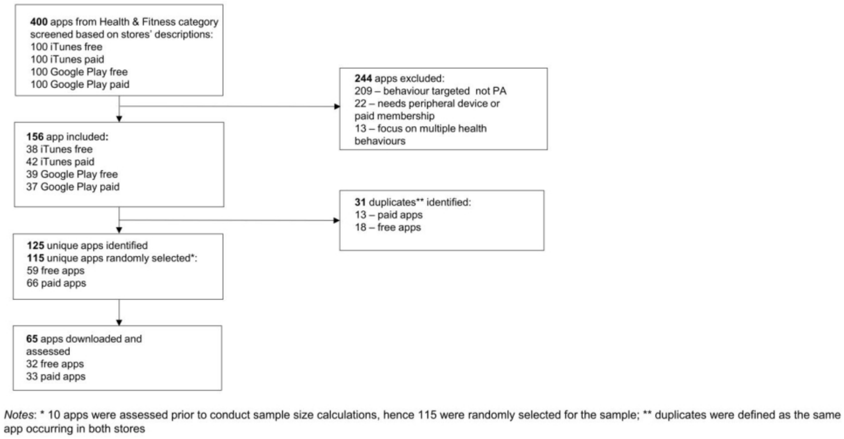

Figure 1: Flow chart of the apps included in the analysis

140x78mm (300 x 300 DPI)

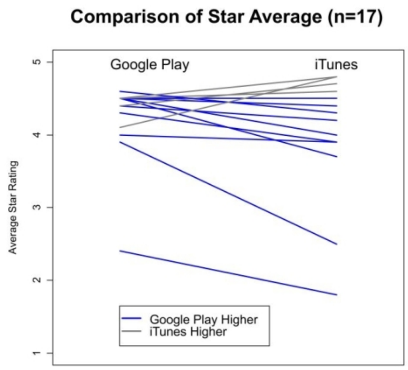

Figure 2: The comparison of star average between the same apps that existed in both stores (n=17)

140x78mm (300 x 300 DPI)

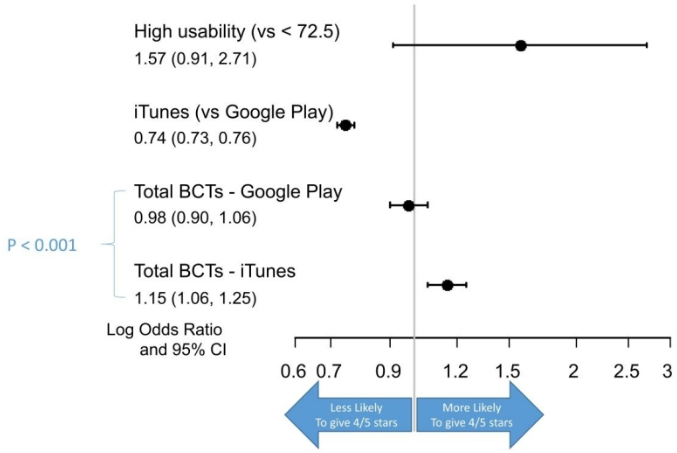

Figure 3: ORs for the associations between the number of BCTs and a 4- or 5- star rating

140x78mm (300 x 300 DPI)

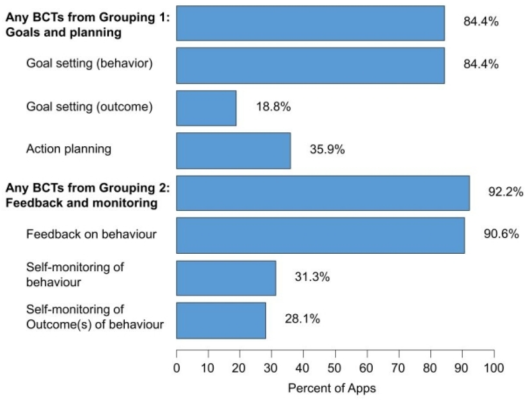

Figure 4: Frequency of individual BCTs within the two Groupings of self-regulatory BCTs

140x78mm (300 x 300 DPI)

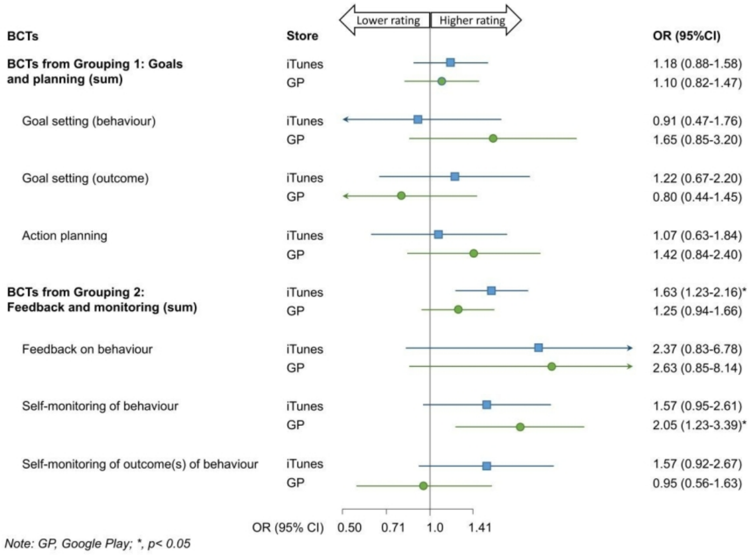

140x78mm (300 x 300 DPI)

Supplementary file 1: Definition of PA app features and the frequency of occurrence

	Features	Definitions/examples
1.	PA EDUCATION IN A VARIETY OF FORMATS	Information, instruction, guidance on PA, e.g., written material, image, audio, video instructions
2.	AUTOMATIC TRACKING	Any data automatically recorded by the app, e.g., step count, duration, PA sessions completed
3.	LOGS	Any data inputted by the user, e.g., calories, weight, gym sessions completed
4.	REPORTS	Any reports based on automatically recorded or user-inputted data, e.g., graphs of the number of steps completed in a week, summaries
5.	GAMIFICATION	Gamification was defined as “the use of game design elements in non-game contexts”*, e.g., provision of, medals, badges, points etc.
6.	NOTIFICATIONS	Messages reminding the user to compete a PA session
7.	IN-APP COMMUNITY	Option to communicate with other users, e.g., forums for sharing PA advice; feed with user reporting on PA sessions completed
8.	SHARING ON SOCIAL MEDIA	Option to post information on completed PA session on social media
9.	INTEGRATION WITH OTHER APPS OR	Option to use or connect with other apps or devices, e.g., Apple Health, music library, sensors such as heart rate monitor
10.	TAILORING of PA	Option to change the default setting of PA sessions, e.g., duration, pace, timing of intervals

*Definition of gamification from: Huotari, K. and Hamari, J. Defining gamification: a service marketing perspective. In *Proceeding of the 16th international academic MindTrek conference;* 2012. ACM. doi: 10.1145/2181037.2181040

App feature (n,%)		
PA education	Yes	44 (67.7)
	No	21 (32.3)
Automatic Tracking	Yes	61(93.9)
	No	4(6.2)
Logs	Yes	24(36.9)
	No	41(63.1)
Reports	Yes	45(69.2)
	No	20(30.8)
Gamification	Yes	27(41.5)
	No	38(58.5)
Reminders	Yes	31(47.7)
	No	34(52.3)
In-app community	Yes	21(32.3)
	No	44(67.7)
Sharing on social media	Yes	32(49.2)
	No	33(50.8)
Integration with other apps/devices	Yes	35(53.8)
	No	30(46.2)
Tailoring of PA	Yes	57(87.7)
	No	8(12.3)

For peer review only - http://bmjopen.bmj.com/site/about/guidelines.xhtml

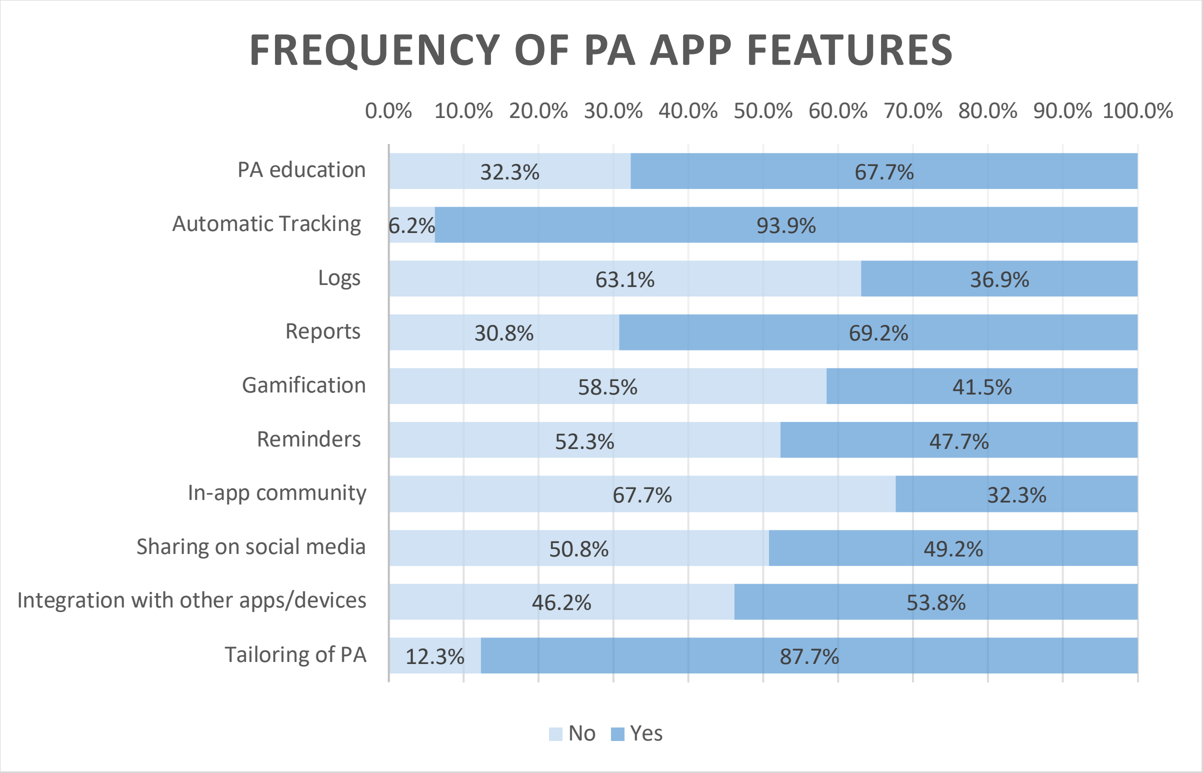
FREQUENCY OF PA APP FEATURES
0.0%
10.0%
20.0%
30.0%
40.0%
50.0%
60.0%
70.0%
80.0%
90.0%
100.0%
PA education
32.3%
67.7%
Automatic Tracking
6.2%
93.9%
Logs
63.1%
36.9%
Reports
30.8%
69.2%
Gamification
58.5%
41.5%
Reminders
52.3%
47.7%
In-app community
67.7%
32.3%
Sharing on social media
50.8%
49.2%
Integration with other apps/devices
46.2%
53.8%
Tailoring of PA
12.3%
87.7%
No
Yes

Supplementary file 2: Sensitivity analysis including only those BCTs for which there was high level of confidence in the presence of the BCT (++)

Variable	App store	OR (CI)	App store	OR (CI)
Group 1: Goals and planning	iTunes	1.16 (0.87-1.55)	Google Play	1.11 (0.83-1.49)
1.1. Goal setting (behavior)	iTunes	0.91 (0.47-1.76)	Google Play	1.65 (0.85-3.20)
1.3. Goal setting (outcome)	iTunes	1.18 (0.64-2.18)	Google Play	0.81 (0.44-1.49)
1.4. Action planning	iTunes	1.07 (0.63-1.84)	Google Play	1.42 (0.84-2.40)
Group 2: Feedback and monitoring	iTunes	1.62 (1.22-2.13)*	Google Play	1.25 (0.95-1.64)
2.2. Feedback on behaviour	iTunes	2.07 (1.02-4.22)*	Google Play	1.76 (0.84-3.68)
2.3. Self-monitoring of behaviour	iTunes	1.57 (0.95-2.61)	Google Play	2.05 (1.23-3.39)*
2.4. Self-monitoring of outcome(s) of behaviour	iTunes	1.61 (0.93-2.77)	Google Play	0.98 (0.57-1.69)

Feedback on behavior crosses into significance for iTunes. Otherwise, the results are very consistent with the original findings.

BMJ Open

Relationship between popularity and the likely efficacy: an observational study based on a random selection on top-ranked physical activity apps

Journal:	*BMJ Open*
Manuscript ID	bmjopen-2018-027536.R2
Article Type:	Original research
Date Submitted by the Author:	24-Sep-2019
Complete List of Authors:	Bondaronek, Paulina; University College London, eHealth Unit, Research Department of Primary Care and Population Health<br>Slee, April; University College London, Research Department of Primary Care and Population Health<br>Hamilton, Fiona; University College London, eHealth Unit, Research Department of Primary Care and Population Health<br>Murray, Elizabeth; University College London, Primary Care and Population Health
**Primary Subject Heading**:	Public health
Secondary Subject Heading:	Health services research, Health policy, Sports and exercise medicine
Keywords:	mHealth, PUBLIC HEALTH, Physical Activity, Behaviour Change, Intervention

SCHOLARONE™
Manuscripts

Relationship between popularity and the likely efficacy: an observational study based on a random selection on top-ranked physical activity apps

Bondaronek Paulina [1], Slee April [2], Hamilton Fiona [1], Murray Elizabeth [1]

1 - eHealth Unit, Research Department of Primary Care and Population Health, University College London

2 - Research Department of Primary Care and Population Health, University College London

Corresponding author: Paulina Bondaronek, eHealth Unit, Department of Primary Care & Population Health, University College London, Upper Third Floor, UCL Medical School (Royal Free Campus) Rowland Hill Street, London NW3 2PF; email: p.bondaronek@ucl.ac.uk; phone: 07742966769

Word count: 4432

For peer review only

ABSTRACT

Objectives: To explore the relationship between popularity of mobile application (apps) for physical activity (PA) and their likely efficacy. The primary objective was to assess the association between app popularity (indicated by user ratings) and likely efficacy (indicated by the number of Behaviour Change Techniques, BCTs, present). The secondary objective was to assess the relationship between user ratings and those BCTs that have been shown to be effective in increasing PA.

Design: Observational study

Methods: 400 top-ranked free and paid apps from iTunes and Google Play stores were screened, and were included if the primary behaviour targeted was PA and they had stand-alone functionality. The outcome variable of user rating was dichotomised into high (4,5 stars) or low (1,2,3 stars) ratings.

Setting: iTunes and Google Play app stores

Participants: No individual participants but the study utilised use-led rating system in the app store

Primary and secondary outcome measures: BCTs and user rating

Results: Of 400 apps, 156 were eligible and 65 apps were randomly selected, downloaded and assessed by two reviewers. There was no relationship overall between star ratings and the number of BCTs present, nor between star ratings and the presence of BCTs known to be effective in increasing PA. App store was strongly associated with star ratings, with lower likelihood of finding 4 or 5 stars in iTunes compared to Google Play (Odds ratio, OR: 0.74 95% CI: 0.73-0.76, p< 0.001).

Conclusions: The findings of this study suggest that popularity does not necessarily imply the likelihood of effectiveness. Hence, public health impact is unlikely to be achieved by allowing market forces to "prescribe" what is used by the public.

For peer review only

Strengths and limitations of this study

- First study to assess the relationship between popularity of physical activity apps and their likely efficacy in two major app stores
- A systematic approach to sample identification and assessment including a standardised assessment tool used to characterise behaviour change interventions
- Sample identified and assessed by two independent reviewers
- It is unknown what variables comprise the ranking algorithm from which the sample was derived
- It is possible that user ratings, as expressed by the stars assigned to the apps, can relate to different aspects of the app content and functionalities

Keywords: mHealth, PUBLIC HEALTH, Physical Activity, Behaviour Change, Intervention

For peer review only

INTRODUCTION

The accessibility, convenience, and wide reach of apps create new avenues for health behaviour change on a large scale. Out of the total 325,000 health apps available on the market in 2017 [1] the largest app groups were fitness apps. The rapidly increased market supply of the apps reflects public demand for the new means of engaging in health behaviours. In 2015, 34% percent of mobile phone owners had at least one health-related app downloaded on their mobile phone [2]. Yet, 28.7% of adults in England are inactive [3]. This discrepancy may be explained by the difference between the intention to increase PA and the actual engagement in the behaviour, i.e, the intention-behaviour gap[4].

Researchers assessing the relationship between the popularity of apps and their quality has found mixed results. Azar, et al. [5] found that apps for weight management that were of higher quality, defined in the their study as inclusion of the constructs from four behaviour change theories, were not among the highest ranked apps the app stores. Similarly, apps that had higher download rates or higher ranking were associated with less adherence to guidelines in smoking cessation apps [6]. On the other hand, Pereira-Azevedo, et al. [7] reviewed the descriptions of 129 urology apps in the Google Play app store and found that higher download rates were associated with expert involvement in the development of the apps. These studies targeted different health conditions and behaviours from physical activity, the subject of this study, and used different definitions of quality, such as consistency with behaviour change theory, expert involvement, and adherence to guidelines, hence their findings need to be interpreted with caution. None of these studies used behaviour change theory to systematically assess the content of the apps in terms of their likely efficacy and how it relates to app popularity.

The Behaviour Change Technique (BCT) Taxonomy [8] is a systematic method used to specify the content of behaviour change interventions [9]. The Taxonomy has also been used to quantify the inclusion of behaviour change theory in interventions, including apps [10-12]. For example, Bardus, et al. [10] extracted BCTs from 23 apps aimed at weight management. They used the Mobile App Rating Scale (MARS) to assess the quality of apps [13] and showed a positive association between the number of BCTs and the MARS subsets: Engagement, Functionality, and Aesthetics, as well as the overall quality of app score. This may suggest that the inclusion of BCTs may be related to the quality of the apps, as assessed by MARS.

However, the same authors found no association between user ratings and either BCTs or the quality indicators on the MARS scale suggesting that apps that were highly rated were not of high quality. Similarly, Crane, et al. [11] showed that only 1 BCT, "prompt review of goals", was associated with user ratings in alcohol reduction apps. The authors concluded that there was little association between the mention of theory, BCTs inclusion and popularity of the alcohol reduction apps. These findings indicate that further work is needed to explore whether popular apps are those that are of high quality and are likely to be effective. This has relevance for public health policy as the combination of popularity with likely efficacy would suggest that apps have a potential role in promoting public health, whereas a disconnect between popularity and likelihood of efficacy would raise questions about leaving the market to guide user choices in app downloads.

Systematic reviews and meta-analyses have shown that self-regulation techniques, such as goal setting, monitoring, and feedback are effective in increasing PA [14-17] behaviour and they may have cumulative effects. For example, Michie, et al. [15] showed that self-monitoring with other self-regulatory BCTs were more effective in increasing PA than using one of those BCTs in isolation. Self-regulation has been acknowledged as important constructs in behaviour change theories, e.g. in Control Theory [18] and Self-Regulation Theory [19]. Hence, the presence of these BCTs can be used as an indicator for quality of those apps and a proxy measure of their likely efficacy.

For peer review only - http://bmjopen.bmj.com/site/about/guidelines.xhtml

Aim and objectives

The aim of this study was to explore the relationship between the user ratings as a marker of popularity of apps and the inclusion of BCTs as a marker of likely efficacy in publicly available PA apps. Specifically, the primary objective was to assess the association between user ratings and the number of BCTs included in the sample of popular PA apps. The secondary objective assessed the inclusion of BCTs shown to be effective in increasing PA behaviour, in particular BCTs related to self-regulation of behaviour.

METHODS

Design

The study used a random sample of popular apps to determine the association between user ratings and the presence of BCTs. Descriptive data included the cost and size of the app; the number and distribution of star ratings; and the presence of BCTs.

Data sources and collection

The sample included popular PA apps identified from 400 top-ranked free and paid apps from the Health and Fitness category (100 iTunes free + 100 iTunes paid + 100 Google Play free + 100 Google Play paid) available in the UK version of iTunes and Google Play app stores on 17/10/16. Apps were included if (i) the primary behaviour targeted was PA; (ii) they had stand-alone functionality. Apps that specifically targeted children were excluded. These apps had to include phrases that suggested clear targeting of children. For example, "Yoga for kids", "Workout for kids", and "Fun fitness for kids", "Toddler activities" would have been excluded. The rationale for excluding apps aimed at children was (i) children may not have access to iTunes or Google Play accounts; (ii) ratings may reflect the parental ratings rather than the children's and (iii) the determinants of PA in children differ from those for adults, with family and school-based activities having a major influence [20 21]. However, such apps were not included in the 400 top ranked apps. Two reviewers (PB, GA) reviewed each app, assessed whether specific BCTs were present, and extracted relevant data. If the apps existed in both stores, then the reviewers only downloaded and assessed apps in iTunes. The detailed process of app identification and sample description is provided in [22] . Based on the number of ratings, the proportion, and the distribution of star ratings we were able to reconstitute individual level response data within each store.

Data extraction

BCTs extraction as a measure of likely efficacy

The presence or absence of 93 BCTs in the BCT Taxonomy v1 as described by Michie, et al. [8] were classified using 3 categories: absent, appears to be present but evidence is not clear (+), present beyond doubt and evidence clear (++). The presence of self-regulation BCTs, associated with PA intervention effectiveness [14-17] [16]was classified using the BCT Taxonomy Grouping 1: Goal and planning, and Grouping 2: Feedback and monitoring as these groups reflect the self-regulation BCTs in the Taxonomy.

User ratings

We extracted the star ratings (1-5 stars) assigned to the apps in both stores and the number of ratings assigned to each app. The average star rating was calculated by summing the number of stars awarded across all users and dividing them by the number of users that submitted the rating. When an app appeared in both stores, a weighted average of the star ratings for each store was calculated based on the relative proportion of the ratings in each store. This algorithm is equivalent to summing the number of stars awarded by all users in both stores and then dividing them by the total number of reviews across both stores. This calculation weights users equally regardless of the platform used to access the app. The variable was dichotomised into high (4,5 stars) or low (1,2,3 stars) ratings.

For peer review only - http://bmjopen.bmj.com/site/about/guidelines.xhtml

Variables determined a priori potentially to affect the relationship between BCTs and higher or lower ratings were app store (iTunes or Google Play), number of features, whether the app was free or required payment, size (in megabytes, MB) and usability.

Number of features

Health apps use technology-enhanced features to deliver BCTs in order to influence behaviour. The apps were categorised according to the features offered by the app, e.g., PA tracking, reminders, app community, data visualisation, etc.). To the authors' best knowledge, at the time of writing, there was no standardised list of features that are commonly used in PA apps. The first ten apps extracted were used to compile a list of PA app features. The list was continuously updated throughout the app extraction process in order to accommodate for new features that were found in the PA app sample. The features were extracted by two reviewers (PB, GA and any discrepancies were resolved by comparing the results of the extraction and reaching consensus).

Usability

Usability was assessed using the System Usability Scale, SUS, [23] which consists of 10 items ranked on a 5-point Likert scale, from "strongly disagree" to "strongly agree". To make the wording of the scale more applicable to the study, two changes were made: 1) the wording of the 8th statement was changed from "cumbersome" to "awkward" as recommended [24-26]; 2) the word "system" was replaced by "app". SUS yields scores from 0-100 where higher scores indicate greater usability. The interpretation of the SUS score used the thresholds proposed and validated by Bangor, et al. [24] with 72.5 described as good usability.

Data analysis

Agreement statistics including the prevalence and bias adjusted kappa statistic, PABAK [27] and unadjusted kappa [28] were calculated. Disagreements were then resolved by discussions among the two reviewers and consultation with other authors if unresolved.

The number of BCTs in the apps was summarized using the mean, standard deviation, median, 25th and 75th percentiles, and the maximum and minimum. Similar statistics were used to summarize user ratings, cost, size, and SUS score. The summary descriptive tables were presented for free and paid apps separately and in total as app stores have separate rankings based on the cost.

The pre-specified primary analysis was based on a linear regression of the number of BCTs on star average as this continuous outcome required the smallest sample of apps for adequate statistical power. Logistic regression to determine the relationship between the number of BCTs and the odds of high and low star rating was the pre-specified secondary analysis. We modelled a higher (versus lower) star rating as the event.

Sample size calculations

The sample size calculation was based on pilot sample of 10 apps (5 paid from iTunes, 5 free from Google Play) selected from the 400 app identified. Apps were sorted in order of store rankings. From iTunes, 38 potentially eligible paid apps were identified and every 8th app was included in the pilot sample (N = 38/5 =7.6= ~8). From Google Play, 55 potentially eligible free apps were identified and every 11th app was included in the pilot sample (N = 55/5 =11). If a sample app was downloaded and found to be ineligible, the next lowest ranked app was used instead. Three apps from Google Play and none from iTunes were found to be ineligible and replaced.

A pilot study based on the 10 apps suggested that mean star ratings were normally distributed. Hence, a sample size calculation was undertaken using mean ratings as a continuous measure in a linear model. Based on this framework, a sample size of 51 apps would provide 90% power to detect a change of 0.11 for each additional BCT at 5% significance level (type I error rate). A sample size of 65 apps was selected to account for any randomly selected apps that might not fulfil the inclusion criteria once downloaded. However, the pilot sample was not sufficiently large enough to show that there was a skew in star average for the complete sample once data were extracted, which would preclude the

For peer review only - http://bmjopen.bmj.com/site/about/guidelines.xhtml

use of linear models. The difference in star rating by store was also unknown at the time of study planning, and hence the app store interactions were a post-hoc addition to this plan. A retrospective power analysis was conducted. Based on simulations, this study had 97% power to detect an OR >= 1.2 with 64 apps. Since we had high power and did not see a significant result, this suggests that the true increase in the chance of a star rating of 4 or 5 with each additional BCT has an OR of < 1.2.

As indicated in the analysis plan, star ratings of 4 and 5 were classified as high ratings, while ratings of 1, 2 and 3 were classified as low ratings. We used the user ratings data from each store in regression models with a random effect to account for store differences. Modelling proceeded in a series of steps. First, we tested a small set of variables identified in the analysis plan for the relationship to star rating (cost, usability, number of features, size) by including each of these as fixed covariates in univariate logistic regression models with the outcome of high versus low ratings. Any variables significant at $p \leq 0.1$ were included in models examining BCTs. App store and the app store-by-BCT interaction terms were included in each model to reflect that the clientele for iTunes and Google Play may be different, and that these differences may impact the relationship between BCTs and star ratings. Weighted logistic regression (by the number of responses for each app) was used with a random intercept term for app, reflecting that the apps selected for analysis were a random sample of available PA apps, and to control for correlation when the same app was reviewed in both stores. Analyses were performed using SAS version 9.3 and R version 3.3.3.

Sensitivity analysis

Sensitivity analyses included a revision of the number of BCTs including only those that were present beyond any doubt (++) [29], and dichotomising user ratings into 5 stars versus < 5 stars.

Patient and Public Involvement

This research was conducted without patient involvement.

RESULTS

Sample

Out of 400 apps, 244 apps were excluded (see Figure 1). Of the remaining 156 PA apps, 31 were duplicates in that the same app appeared in both iTunes and Google Play. Thus, 125 unique apps were eligible for random selection; 65 unique apps were randomly selected for analysis including 32 free apps and 33 paid apps. One app, Break, was excluded from analyses of user reviews as the data on user ratings were not available due to the small number of ratings, resulting in a final sample of 64 PA apps. These apps collectively received more than 2.8 million user ratings. An individual-level data of the characteristics of each app is provided in Bondaronek, et al. [22].

Figure 1: Flow chart of the apps included in the analysis

<Figure 1 separate file>

There was substantial agreement between the reviewers in the assessment of BCTs (PABAK =0.94, 95% CI 0.93 to 0.95, Kappa =0.78, 95% CI 0.75 to 0.81).

Characteristics of the apps are displayed in Table 1. The number of BCTs and features were approximately normally distributed, with the mean number of BCTs being 7.1 (SD= 3.0) and the mean number of features being 5.8 (SD=2.1) (see Supplementary file 1 for list of PA app features and frequency of occurrence). Star ratings, size, SUS score and the cost variables were skewed. The median star rating was 4.5 (IQR 1.9 – 4.9) and the median SUS score was 86.3 (IQR 75.00 – 91.88). Among paid apps, the median cost was £2.40 (IQR 1.78 – 2.99).

For peer review only - http://bmjopen.bmj.com/site/about/guidelines.xhtml

For peer review only

Table 1: Characteristics of PA apps included in the sample

		Free (n=32)	Paid (n=32)	Total (n=64)
Number of BCTs	Mean ± SD	6.56 ± 2.99	7.56 ± 2.87	7.06 ± 2.95
	Median	7.00	8.00	8.00
	25, 75%tile	5.00, 8.00	6.00, 10.00	5.00, 9.00
Star average*	Mean ± SD	4.26 ± 0.65	4.35 ± 0.45	4.30 ± 0.56
	Median	4.50	4.49	4.50
	25, 75%tile	4.30, 4.60	4.29, 4.60	4.30, 4.60
Number of Features	Mean ± SD	5.94 ± 2.17	5.72 ± 1.95	5.8 ± 2.05
	Median	7.00	8.00	6.00
	25, 75%tile	4.00, 8.00	5.00, 8.00	4.00, 8.00
Size (MB)*	Mean ± SD	45.53 ± 40.85	61.51 ± 47.22	53.52 ± 44.53
	Median	32.45	53.97	39.64
	25, 75%tile	16.82, 62.27	28.92, 85.02	25.52, 79.50
Usability (SUS)	Mean ± SD	81.25 ± 12.64	85.23 ± 12.02	83.24 ± 12.40
	Median	85.00	87.50	86.25
	25, 75%tile	71.88, 91.25	78.75, 93.75	75.00, 91.88
Cost (GBP) *	Mean ± SD		3.00 ± 2.12	
	Median	N/A	2.35	N/A
	25, 75%tile		1.78, 2.99	

*weighted average of iTunes and Google Play; MB: megabytes; GBP: British pounds, N/A, not applicable

User ratings

In total, 2,819,469 user ratings of the 64 apps were used in the analysis. Among these, 88.5% were 4 or 5 star reviews and 11.5% were 1, 2 or 3-star reviews. Among the covariates considered for model inclusion, good usability increased the chance of a 4 or 5 star rating compared to poor usability (OR: 1.66, 95% CI 0.96 to 2.89, p= 0.071). This covariate was included in all subsequent models. App store was strongly associated with star ratings, with lower likelihood of finding 4 or 5 stars in iTunes compared to Google Play (OR: 0.74 95% CI: 0.73 to 0.76, p< 0.001).

Further investigation of the difference between the stores and user ratings showed that there was 37% higher likelihood of awarding 4 or 5 stars in Google Play compared to iTunes (95%CI 35% to 41%, p < 0.001). A similar pattern can be seen comparing the weighted average within-app star rating across stores. There were 17 apps that were sold in both stores (Figure 2). Analysis was conducted to assess whether the proportion with the high star ratings in Google

For peer review only - http://bmjopen.bmj.com/site/about/guidelines.xhtml

Play exceeds what would have been found by chance. If there were no relationship between store and star rating, we would expect the star rating in iTunes to exceed the star rating in Google Play for about 50% of apps (coin flip). Mean star rating in Google Play exceeded the rating in iTunes in 13/17 apps (76.5%, 95% CI: 64.2 to 86.2, p<0.0001). Since the analysis was significant, we can rule out that the difference between the user ratings in the stores was due to chance. Cost, size and number of features were not related to star ratings.

Figure 2: The comparison of star average between the same apps that existed in both stores (n=17)

<Figure 2 separate file>

Primary analysis

The primary analysis of the association between user ratings and the overall number of BCTs included in each app found no relationship between number of BCTs and star rating (OR: 1.05, 95% CI: 0.97 to 1.14, p =0.236). Subgroup analysis showed that in iTunes only, a higher star rating was associated with the total number of BCTs (OR: 0.72, 95% CI: 0.71 to 0.74, p< 0.001).

Based on the model containing the store interaction (Figure 3), in iTunes there was an association with each additional BCT corresponding to 15% increase in the likelihood of a higher star rating (OR: 1.15, 95% CI: 1.06, 1.25), there was no association between the number of BCTs and Google Play. Usability was not significant in the multivariate model (OR 1.57, 95% CI: 0.91, 2.71)

Figure 3: ORs for the associations between the number of BCTs and a 4- or 5- star rating

<Figure 3 separate file>

Secondary objective

The secondary analysis examined the associations between a 4- or 5- star user rating and the inclusion of BCTs that have been shown to be effective in increasing PA behaviour.

The frequency of the BCTs associated with efficacy for increasing PA behaviour is presented in Figure 4.
The BCT grouping 1 (Goal and planning) was represented in 84.4% of evaluated apps. The most common of these was goal setting for behaviours (84.4%), followed by action planning (35.9%) and goal setting for outcomes (18.8%). The BCT grouping 2 (Feedback and monitoring) was included in 92.2% of evaluated apps. Feedback on behaviour was included in nearly all apps (90.6%), while self-monitoring of behaviour and of outcomes were incorporated in about a third of apps.

Figure 4: Frequency of individual BCTs within the two Groupings of self-regulatory BCTs

<Figure 4 separate file>

The number of Grouping 1 BCTs had no impact on star rating in either store (Figure 5). Among iTunes users, each additional BCT from Grouping 2 increased the chance of a high star rating by 63% (OR: 1.63 95% CI: 1.23-2.16*). There* was no association between the number of BCTs and star rating among Google Play users. Self-monitoring of behaviour was associated with higher ratings in Google Play (OR: 2.05, 95% CI: 1.23-3.39)

For peer review only - http://bmjopen.bmj.com/site/about/guidelines.xhtml

Figure 5: ORs for the associations between BCTs that have been shown to be effective and a 4- or 5- star rating
<Figure 5 separate file>

Sensitivity analysis

The sensitivity analyses including only those BCTs that were classified as "present beyond all reasonable doubt" [29] showed that for iTunes, the BCT 2.2 Feedback on behaviour crossed into significance. Otherwise, the results are consistent with the original findings. The results of the sensitivity analysis are available in the Supplementary file 2.

DISCUSSION

Statement of principal findings

The aim of this study was to assess the relationship between the popularity of publicly available PA apps (assessed through user ratings) and their likely efficacy (assessed through the inclusion of BCTs). Overall, for both app stores, there was no association between popularity and likely efficacy as indicated by the overall presence of BCTs and the BCTs known to be effective in increasing PA. However, users in each app stores differed and there was an association between the number of BCTs and high user rating in iTunes but not in Google Play.

Strengths and weaknesses of this study

The main strength of the study includes the systematic assessment of the app content conducted by two reviewers. The sample was identified from the most popular publicly available apps from two major app distribution platforms. The use of BCT taxonomy provides a standardised assessment tool, which has been utilised in other studies assessing the content of apps [12 30-32]. Second, Guzman [33] argued that the star rating represents an average score for the whole app that combines both positive and negative evaluations aggregated across users. The study, however, used rater-level data which included individual ratings from 2.8 million users. These large numbers mitigate the problem posed by averaging the star rating across users.

This study has some important limitations. First, the main limitation of the study relates to the variables used in this study. It is possible that user ratings, as expressed by the stars assigned to the apps, can relate to different aspects of the app functioning and content. There is evidence suggesting that app reviews tend to occur near a new release which can suggest that the ratings may include comments on the specific updates of the software [34]. In addition, the possibility that user ratings were influenced by fake reviews cannot be excluded [35 36]. However, the user rating was considered the most appropriate measure to explore since it represents a user-led feedback that reflects user experience. Similarly, the choice of the BCTs as an approximation for likely efficacy was selected because studies assessing the efficacy of the apps on the market are scarce [37-39]. Second, the ranking algorithm from which the sample was derived is unknown. Hence, this lack of transparency prevented evaluation of how the calculation of rank might have influenced the app selection. However, apps appear in rank order by default in app stores hence the rank affects what users are seeing. As the aim of the study was to assess the most popular apps, the choice of highly ranked apps was considered the most appropriate for the context of this research. Third, Google Play market tend to have more ratings than iTunes because the process of app review is more complex in the latter [40]. This was addressed in the study by using the weighted averages of the ratings across the stores (for the summary of the app characteristics), and by controlling for store in the regression models. While there is a difference in the app review process in both app stores, feedback from both stores should be recognised as valid and important. Fourth, while we inspected the apps to ensure that the duplicate apps were similar in both stores, there could be differences in the functionality of the apps between the app stores that we could not see. Fifth, failure to detect the skew in the original primary outcome used to power the study is a limitation. However, a retrospective power analysis showed we had high power to detect an OR of 1.2

For peer review only - http://bmjopen.bmj.com/site/about/guidelines.xhtml

and did not find significant result, hence the true OR is likely to be < 1.2. Lastly, the sample identification was obtained in October 2016 reflecting an extract of the state of the market at the time.

Strengths and weaknesses in relation to other studies, discussing particularly any differences in results

This study supports the findings of previous research in apps targeting weight management [5 10], smoking cessation [6] and alcohol reduction [11] which showed that apps that were highly rated, highly ranked or highly downloaded were not necessarily of high quality. In addition, the inclusion of BCTs that have been shown to be effective in increasing PA, i.e., the self-regulation strategies, were also not associated with higher ratings and this results supports the similar findings of Bardus, et al. [10]. No association between the behaviour change theory content and user ratings suggests other factors may be contributing to the apps popularity. High quality graphic design, visual appeal and ease of use are more likely to attract potential customers to download and engage with the app[41]. In addition, the promotion of the apps can play a role in the download rates. The strength of this study, in comparison to other handful research exploring this association, is the use of logistic regressions, including the analysis of the potential confounding factors, provides a strong evidence for the association, if such was existing.

The finding of an association between user rankings and overall number of BCTs in one store (iTunes) but not the other (Google Play) is new and one that has not been found in previous studies. There are various possible explanations for this finding. Market researchers suggest possible differences in the population of users of each store [42 43]. For example, iPhone users might be more affluent, they engage with their device for longer, make more purchases with their phone, and are more loyal to their brand [44]. In addition, there are differences in the review and approval processes between the two stores which could have influence the user ratings [45]. Future studies should explore this observation further, and determine whether it holds for health apps targeting different behaviours.

Meaning of the study: possible mechanisms and implications for clinicians or policymakers

In this study, we showed no evidence of association between popularity and likely efficacy. The implication of this study is that the popularity of these apps is not a sufficient filter to distinguish the apps that might have higher potential to have an impact on the potential users. Hence, we suggest that, at present, allowing the commercial market to determine which PA apps are downloaded is unlikely to be an effective method of public health promotion in terms of increasing overall levels of PA.

Based on the findings, we suggest some implications for public health policy. Apps aimed to increase PA represent the largest category in the two major app stores [46] which illustrates public demand for engaging in PA. The lack of quality in those apps indicates a missed opportunity to increase health at the population level. Initiatives to identify and promote high quality apps are in development, e.g., the NHS Apps Library [47]. However, there is an urgent need to evaluate the effectiveness of these apps with potential users.

Unanswered questions and future research

Further research is needed to understand which BCTs, and in which combination, are most effective in increasing PA when delivered via an app; how these BCTs can best be delivered; and how to combine features which promote efficacy with those that promote popularity. Last, the differences in iTunes and Google Play users is an unexpected finding, and not one that the study set out to identify, (i.e. not an a priori hypothesis). Future researchers should be aware of the potential differences between iTunes and Google Play users and ensure research is carried out on both platforms.

Conclusion

To date, this is the first study to assess the association between popularity (measured using user ratings of the apps) and likely efficacy (measured using the inclusion of the BCTs) of publicly available highly-ranked PA apps available in

For peer review only - http://bmjopen.bmj.com/site/about/guidelines.xhtml

the major app stores. No relationship was found between popularity and likely efficacy suggesting that popularity does not assure high quality, and what is liked may not be what is likely to be effective. However, PA apps in this study were highly rated, highly-ranked apps from the major app stores, hence highly visible for the potential user. Hence, promotion of public health is unlikely to be achieved by allowing market forces to determine which PA apps are used. More studies are needed to assess the effectiveness of apps with users in a real-world setting to investigate the app components that are both, effective and valued by the users.

Supplementary files

Supplementary file 1: Definition of PA app features and the frequency of occurrence
Supplementary file 2: Sensitivity analysis including BCTs with only high level of confidence in the presence in the apps (++)

Competing Interests

None declared.

Data Sharing Statement

All data used in the study is available upon request.

Contributorship statement

PB designed the study, collected the data, analysed the data and wrote the manuscript. AS analysed the data and co-wrote the manuscript. EM and FH provided advice on study design, data analysis and interpretation, and contributed to the critical appraisal of the manuscript.

Acknowledgements

We wish to acknowledge Ghadah Alkhaldi (Community Health Sciences Department, College of Applied Medical Sciences, King Saud University, Riyadh, Saudi Arabia) for her help with the data extraction.

Funding

PB is a PhD student funded by the Medical Research Council.

References

1. research2guidance. mHealth App Economics: Current Statu and Future Trends in Mobile Health: research2guidance; 2017 [Available from: https://www.uzelf.org/wp-content/uploads/2017/12/R2G-mHealth-Developer-Economics-2017-Status-And-Trends.pdf accessed 20/09/18 2018.
2. Terry K. Number of health apps soars, but use does not always follow 2015 [Available from: http://www.medscape.com/viewarticle/851226.
3. Public Health England. Public Health Outcomes Framework: Physical Activity 2016 [Available from: http://www.phoutcomes.info/search/physical%20activity#pat/6/ati/102/par/E12000004.
4. Rhodes RE, Dickau L. Moderators of the intention-behaviour relationship in the physical activity domain: a systematic review. *Br J Sports Med* 2013;47(4):215-25.
5. Azar KM, Lesser LI, Laing BY, et al. Mobile applications for weight management: theory-based content analysis. *Am J Prev Med* 2013;45(5):583-9. doi: 10.1016/j.amepre.2013.07.005
6. Abroms LC, Padmanabhan N, Thaweethai L, et al. iPhone apps for smoking cessation: a content analysis. *American journal of preventive medicine* 2011;40(3):279-85.

For peer review only - http://bmjopen.bmj.com/site/about/guidelines.xhtml

7. Pereira-Azevedo N, Osório L, Cavadas V, et al. Expert involvement predicts mHealth app downloads: multivariate regression analysis of urology apps. *JMIR mHealth and uHealth* 2016;4(3) doi: 10.2196/mhealth.5738
8. Michie S, Richardson M, Johnston M, et al. The behavior change technique taxonomy (v1) of 93 hierarchically clustered techniques: building an international consensus for the reporting of behavior change interventions. *Annals of behavioral medicine* 2013;46(1):81-95. doi: 10.1007/s12160-013-9486-6
9. Michie S, Atkins L, West R. The behaviour change wheel. *A guide to designing interventions 1st ed Great Britain: Silverback Publishing* 2014
10. Bardus M, van Beurden SB, Smith JR, et al. A review and content analysis of engagement, functionality, aesthetics, information quality, and change techniques in the most popular commercial apps for weight management. *Int J Behav Nutr Phys Act* 2016;13:35. doi: 10.1186/s12966-016-0359-9
11. Crane D, Garnett C, Brown J, et al. Behavior change techniques in popular alcohol reduction apps: content analysis. *J Med Internet Res* 2015;17(5):e118. doi: 10.2196/jmir.4060
12. Middelweerd A, Mollee JS, van der Wal CN, et al. Apps to promote physical activity among adults: a review and content analysis. *Int J Behav Nutr Phys Act* 2014;11(1):97.
13. Stoyanov SR, Hides L, Kavanagh DJ, et al. Mobile app rating scale: a new tool for assessing the quality of health mobile apps. *JMIR Mhealth Uhealth* 2015;3(1):e27. doi: 10.2196/mhealth.3422
14. Greaves CJ, Sheppard KE, Abraham C, et al. Systematic review of reviews of intervention components associated with increased effectiveness in dietary and physical activity interventions. *BMC public health* 2011;11(1):119.
15. Michie S, Abraham C, Whittington C, et al. Effective techniques in healthy eating and physical activity interventions: a meta-regression. *Health Psychology* 2009;28(6):690. doi: 10.1037/a0016136
16. Conn VS, Hafdahl AR, Mehr DR. Interventions to increase physical activity among healthy adults: meta-analysis of outcomes. *American journal of public health* 2011;101(4):751-58.
17. Pearson ES. Goal setting as a health behavior change strategy in overweight and obese adults: a systematic literature review examining intervention components. *Patient education and counseling* 2012;87(1):32-42. doi: 10.1016/j.pec.2011.07.018
18. Carver CS, Scheier MF. Control theory: A useful conceptual framework for personality–social, clinical, and health psychology. *Psychological bulletin* 1982;92(1):111.
19. Kanfer FH. Implications of a self-regulation model of therapy for treatment of addictive behaviors. Treating addictive behaviors: Springer 1986:29-47.
20. Sallis JF, Prochaska JJ, Taylor WC. A review of correlates of physical activity of children and adolescents. *Medicine and science in sports and exercise* 2000;32(5):963-75.
21. Hesketh KR, O'Malley C, Paes VM, et al. Determinants of Change in Physical Activity in Children 0-6 years of Age: A Systematic Review of Quantitative Literature. *Sports medicine (Auckland, NZ)* 2017;47(7):1349-74. doi: 10.1007/s40279-016-0656-0 [published Online First: 2016/12/19]
22. Bondaronek P, Alkhaldi G, Slee A, et al. Quality of Publicly Available Physical Activity Apps: Review and Content Analysis. *JMIR mHealth and uHealth* 2018;6(3)
23. Brooke J. SUS-A quick and dirty usability scale. *Usability evaluation in industry* 1996;189(194):4-7.
24. Bangor A, Kortum PT, Miller JT. An empirical evaluation of the system usability scale. *Intl Journal of Human–Computer Interaction* 2008;24(6):574-94. doi: 10.1080/10447310802205776
25. Finstad K. The system usability scale and non-native english speakers. *Journal of usability studies* 2006;1(4):185-88.
26. Lewis JR. Psychometric evaluation of the PSSUQ using data from five years of usability studies. *International Journal of Human-Computer Interaction* 2002;14(3-4):463-88. doi: 10.1080/10447318.2002.9669130
27. Byrt T, Bishop J, Carlin JB. Bias, prevalence and kappa. *Journal of clinical epidemiology* 1993;46(5):423-29. doi: 10.1016/0895-4356(93)90018-V
28. Cohen J. Kappa: Coefficient of concordance. *Educ Psych Measurement* 1960;20(37)

For peer review only - http://bmjopen.bmj.com/site/about/guidelines.xhtml

29. Wood CE, Richardson M, Johnston M, et al. Applying the behaviour change technique (BCT) taxonomy v1: a study of coder training. *Translational behavioral medicine* 2014;5(2):134-48.
30. Direito A, Dale LP, Shields E, et al. Do physical activity and dietary smartphone applications incorporate evidence-based behaviour change techniques? *BMC Public Health* 2014;14(1):1.
31. Chen J, Cade JE, Allman-Farinelli M. The Most Popular Smartphone Apps for Weight Loss: A Quality Assessment. *JMIR Mhealth Uhealth* 2015;3(4):e104. doi: 10.2196/mhealth.4334
32. Yang CH, Maher JP, Conroy DE. Implementation of behavior change techniques in mobile applications for physical activity. *Am J Prev Med* 2015;48(4):452-5. doi: 10.1016/j.amepre.2014.10.010
33. How do users like this feature? a fine grained sentiment analysis of app reviews. Requirements Engineering Conference (RE), 2014 IEEE 22nd International; 2014. IEEE.
34. User feedback in the appstore: An empirical study. Requirements Engineering Conference (RE), 2013 21st IEEE International; 2013. IEEE.
35. AppAware: which mobile applications are hot? Proceedings of the 12th international conference on Human computer interaction with mobile devices and services; 2010. ACM.
36. Trusting smartphone apps? To install or not to install, that is the question. Cognitive Methods in Situation Awareness and Decision Support (CogSIMA), 2013 IEEE International Multi-Disciplinary Conference on; 2013. IEEE.
37. Direito A, Carraça E, Rawstorn J, et al. mHealth technologies to influence physical activity and sedentary behaviors: Behavior change techniques, systematic review and meta-analysis of randomized controlled trials. *Annals of behavioral medicine* 2017;51(2):226-39.
38. Buechi R, Faes L, Bachmann LM, et al. Evidence assessing the diagnostic performance of medical smartphone apps: a systematic review and exploratory meta-analysis. *BMJ open* 2017;7(12):e018280.
39. Jake-Schoffman DE, Silfee VJ, Waring ME, et al. Methods for evaluating the content, usability, and efficacy of commercial mobile health apps. *JMIR mHealth and uHealth* 2017;5(12)
40. Xu W, Liu Y. mHealthApps: a repository and database of mobile health apps. *JMIR mHealth and uHealth* 2015;3(1):e28. doi: 10.2196/mhealth.4026
41. Lazard AJ, Mackert MS. E-health first impressions and visual evaluations: key design principles for attention and appeal. *Communication Design Quarterly Review* 2015;3(4):25-34.
42. Götz FM, Stieger S, Reips U-D. Users of the main smartphone operating systems (iOS, Android) differ only little in personality. *PloS one* 2017;12(5):e0176921.
43. Monetate. Ecommerce Quarterly 2016: Mobile ate the world and is still hungry 2017 [Available from: http://info.monetate.com/rs/092-TQN-434/images/Monetate-EQ3-2016.pdf?mkt_tok=eyJpIjoiTURFMFptTTNNR1prWWpRMyIsInQiOiJsaDE4bXF1OTA4R0o4T0xjNGZjXC9JRkcxcHM4WkRTZFZkT2tnWWorMGtIc24zSUFsanN2Z2tOVDJHTDZFc3F1eTZnZEZJaWJsQUxzQWRjN3F6ZXVNc2wrcVREMDFJc3RMQU5yc0NGdXU4OG41Zk9Gdk1OaGZRUXNEcisreWFEczcifQ%3D%3D accessed 21/09/18 2018.
44. Katariya J. Apple Vs Android — A comparative study 2017: AndroidPub; 2017 [Available from: https://android.jlelse.eu/apple-vs-android-a-comparative-study-2017-c5799a0a1683 accessed 2018 2017.
45. Why Do Users Install and Delete Apps? A Survey Study. International Conference of Software Business; 2017. Springer.
46. research2guidance. mHealth app developer economics. The state of the art of mHealth application publishing: research2guidance 2014 [updated 5 May 2016. Available from: http://research2guidance.com/product/mhealth-app-developer-economics-2014/.
47. NHS. NHS Apps Library: NHS; [Available from: https://www.nhs.uk/apps-library/ accessed 16/06/19.

For peer review only - http://bmjopen.bmj.com/site/about/guidelines.xhtml

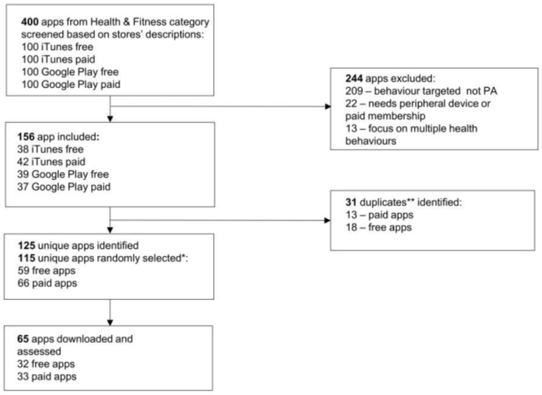

Notes: * 10 apps were assessed prior to conduct sample size calculations, hence 115 were randomly selected for the sample; ** duplicates were defined as the same app occurring in both stores

Figure 1: Flow chart of the apps included in the analysis

140x78mm (300 x 300 DPI)

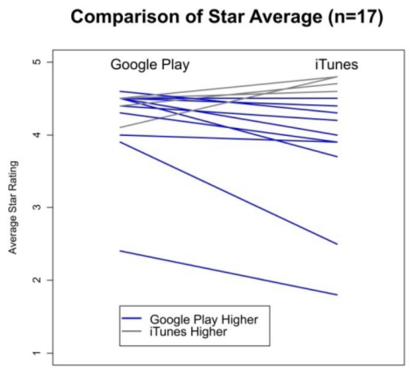

Figure 2: The comparison of star average between the same apps that existed in both stores (n=17)

140x78mm (300 x 300 DPI)

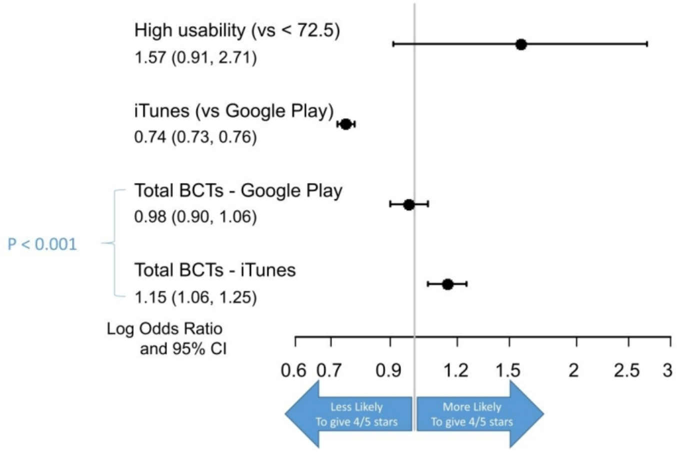

Figure 3: ORs for the associations between the number of BCTs and a 4- or 5- star rating

140x78mm (300 x 300 DPI)

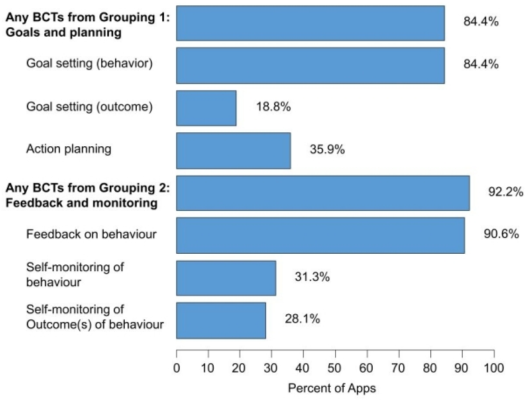

Figure 4: Frequency of individual BCTs within the two Groupings of self-regulatory BCTs

140x78mm (300 x 300 DPI)

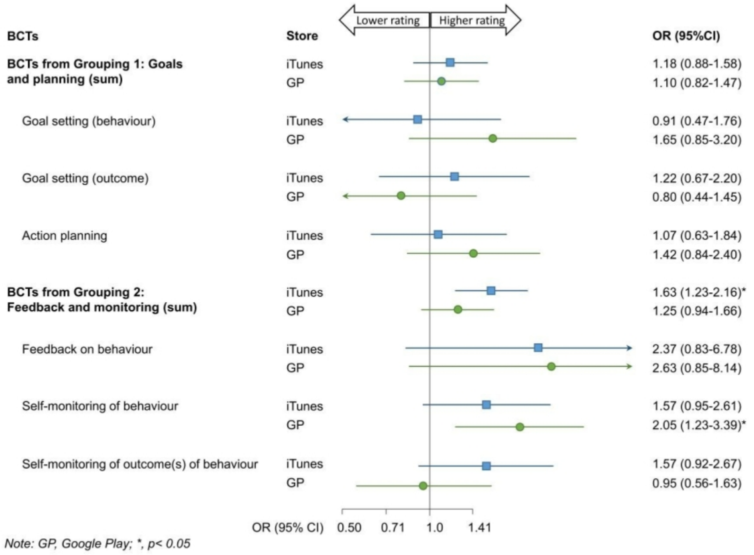

140x78mm (300 x 300 DPI)

Supplementary file 1: Definition of PA app features with the frequency of occurrence and the number of features per app (n= 65)

	Features	Definitions/examples
1.	PA EDUCATION IN A VARIETY OF FORMATS	Information, instruction, guidance on PA, e.g., written material, image, audio, video instructions
2.	AUTOMATIC TRACKING	Any data automatically recorded by the app, e.g., step count, duration, PA sessions completed
3.	LOGS	Any data inputted by the user, e.g., calories, weight, gym sessions completed
4.	REPORTS	Any reports based on automatically recorded or user-inputted data, e.g., graphs of the number of steps completed in a week, summaries
5.	GAMIFICATION	Gamification was defined as “the use of game design elements in non-game contexts”*, e.g., provision of, medals, badges, points etc.
6.	NOTIFICATIONS	Messages reminding the user to compete a PA session
7.	IN-APP COMMUNITY	Option to communicate with other users, e.g., forums for sharing PA advice; feed with user reporting on PA sessions completed
8.	SHARING ON SOCIAL MEDIA	Option to post information on completed PA session on social media
9.	INTEGRATION WITH OTHER APPS OR	Option to use or connect with other apps or devices, e.g., Apple Health, music library, sensors such as heart rate monitor
10.	TAILORING of PA	Option to change the default setting of PA sessions, e.g., duration, pace, timing of intervals

*Definition of gamification from: Huotari, K. and Hamari, J. Defining gamification: a service marketing perspective. In *Proceeding of the 16th international academic MindTrek conference;* 2012. ACM. doi: 10.1145/2181037.2181040

For peer review only - http://bmjopen.bmj.com/site/about/guidelines.xhtml

App feature (n,%)		
PA education	Yes	44 (67.7)
	No	21 (32.3)
Automatic Tracking	Yes	61(93.9)
	No	4(6.2)
Logs	Yes	24(36.9)
	No	41(63.1)
Reports	Yes	45(69.2)
	No	20(30.8)
Gamification	Yes	27(41.5)
	No	38(58.5)
Reminders	Yes	31(47.7)
	No	34(52.3)
In-app community	Yes	21(32.3)
	No	44(67.7)
Sharing on social media	Yes	32(49.2)
	No	33(50.8)
Integration with other apps/devices	Yes	35(53.8)
	No	30(46.2)
Tailoring of PA	Yes	57(87.7)
	No	8(12.3)

For peer review only - http://bmjopen.bmj.com/site/about/guidelines.xhtml

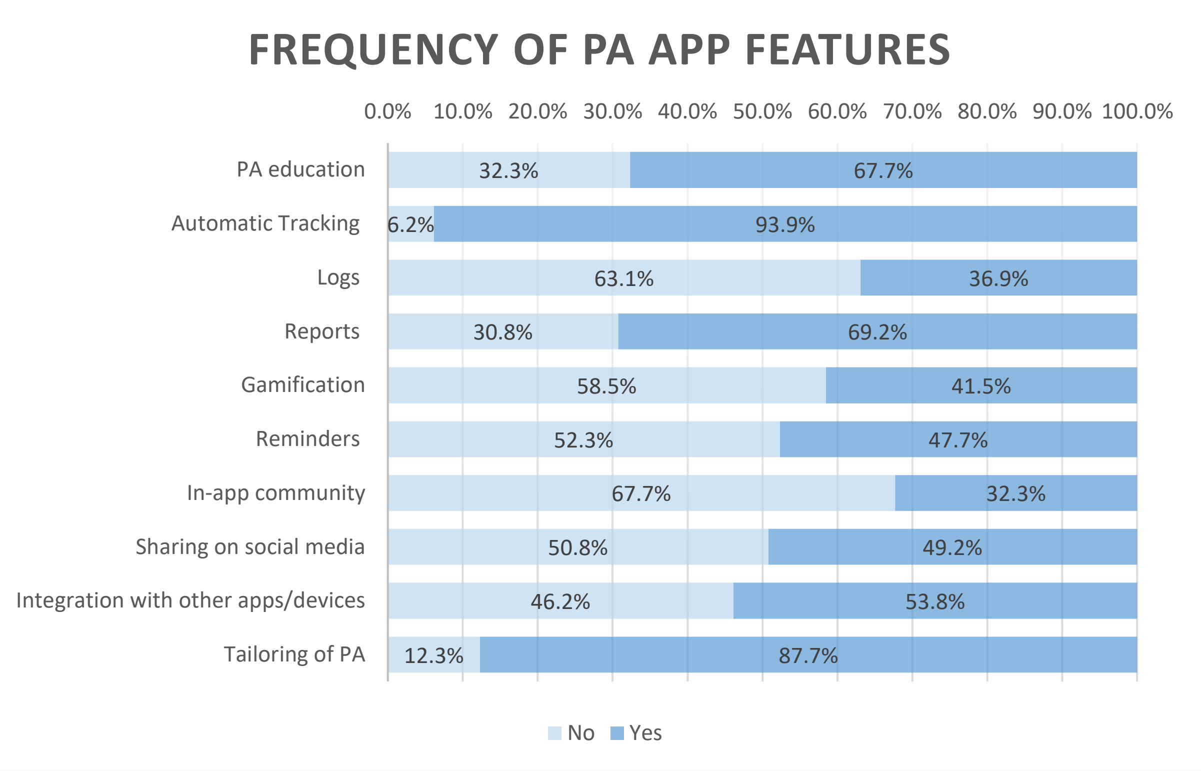

	App name	Number of Features
1.	Fitbit	9
2.	Strava Running and Cycling GPS	8
3.	Pacer – Pedometer plus Weight Loss and BMI Tracker	7
4.	Map My Run – GPS Running & Workout Tracker	9
5.	Adidas train & run	9
6.	Steps Pedometer & Step Counter Activity Tracker	2
7.	7 Minute Workout by Simple Design Ltd	8
8.	Runtastic Running & Fitness	12
9.	Home workout MMA Spartan Free	4
10.	Stepz – Pedometer & Step Counter	9
11.	Interval Timer – Timing for HIIT Training and Workouts	3
12.	Sworkit – Custom Workouts for Exercise & Fitness	7
13.	Fitness & Bodybuilding	5
14.	Daily Workouts FREE	4
15.	30 Day Ab Challenge FREE	6
16.	Runtastic Results: Body Workout Fitness Trainer	9
17.	C25K® - 5K Running Trainer	8
18.	Health Mate – Steps tracker & Life coach	7
19.	One You Couch to 5K	6
20.	Running, Walking and Biking with Endomondo	8
21.	Map My Ride – GPS Cycling & Route Tracker	9
22.	Interval Timer	1
23.	5K Run - Couch to 5K	5
24.	7 Minutes Workout – Women Fitness Exercise Trainer	6
25.	Seconds – Interval Timer	4
26.	Running Distance Tracker +	4
27.	Freeletics Bodyweight – Workout	8
28.	Couch to 10K Running Trainer	8

For peer review only

29.	FitNotes - Gym Workout Log	4
30.	Belly Fat Exercises	1
31.	Belly Fat Workout FREE – 10 Minute Ab Exercises	3
32.	Movesum – Step counter by Lifesum	3
33.	7 Minute Workout Challenge by Fitness Guide Inc	8
34.	Adrian James 6 Pack Abs Workout	6
35.	Full Fitness : Exercise Workout Trainer	5
36.	Runtastic PRO Running and Workout Tracker	10
37.	Couch to 5K® - Running App and Training Coach	9
38.	Adrian James High Intensity Interval Training	6
39.	Running for Weight Loss PRO	9
40.	Fitness Trainer FULL version	4
41.	Instant Fitness : 600+ exercises, 100+ workouts	5
42.	Push ups 0 to 100: push ups challenge trainer pro	7
43.	Couch to 5K Runner, 0 to 5K run training	7
44.	Footsteps – Pedometer	8
45.	iMuscle 2	6
46.	10K Running Trainer Pro	8
47.	Police Fitness – Bleep Test	2
48.	Marathon Trainer - 26.2 42K	8
49.	MapMyFitness+ Workout Trainer	7
50.	5K to 10K	7
51.	7 Minute Workout Pro	7
52.	Chloe Madeley Weights 4 Women	1
53.	10K Pacer: Run pace training. Run faster	8
54.	Starting Strength Official	5
55.	Thor Fitness: 60 Day Bodyweight Workout Routine	3
56.	Half Marathon Trainer 13.1 21K	8
57.	PDC Pole Dance Syllabus	1
58.	MMA Spartan Workouts Pro	5
59.	Get Running (Coach to 5K)	5
60.	WalkJogRun GPS Running Routes	9
61.	Runtastic Road Bike PRO	8
62.	Runtastic Mountain Bike PRO GPS Biking Computer, Trail and Route Tracker	11
63.	Chloe Madeley 15 minute fat loss workout	2
64.	CARROT Fit – 7 Minute Workout, Step Counter Weight Tracker	11

For peer review only

Supplementary file 2: Sensitivity analysis including only those BCTs for which there was high level of confidence in the presence of the BCT (++)

Variable	App store	OR (CI)	App store	OR (CI)
Group 1: Goals and planning	iTunes	1.16 (0.87-1.55)	Google Play	1.11 (0.83-1.49)
1.1. Goal setting (behavior)	iTunes	0.91 (0.47-1.76)	Google Play	1.65 (0.85-3.20)
1.3. Goal setting (outcome)	iTunes	1.18 (0.64-2.18)	Google Play	0.81 (0.44-1.49)
1.4. Action planning	iTunes	1.07 (0.63-1.84)	Google Play	1.42 (0.84-2.40)
Group 2: Feedback and monitoring	iTunes	1.62 (1.22-2.13)*	Google Play	1.25 (0.95-1.64)
2.2. Feedback on behaviour	iTunes	2.07 (1.02-4.22)*	Google Play	1.76 (0.84-3.68)
2.3. Self-monitoring of behaviour	iTunes	1.57 (0.95-2.61)	Google Play	2.05 (1.23-3.39)*
2.4. Self-monitoring of outcome(s) of behaviour	iTunes	1.61 (0.93-2.77)	Google Play	0.98 (0.57-1.69)

Feedback on behavior crosses into significance for iTunes. Otherwise, the results are very consistent with the original findings.
